# Production, isolation, optimization, and characterization of microbial PHA from *Bacillus australimaris*

**DOI:** 10.1038/s41598-025-92146-x

**Published:** 2025-03-11

**Authors:** Rafwana Ibrahim, Jesil Mathew Aranjani, Navya Prasanna, Avirup Biswas, Prasanna Kumar Reddy Gayam

**Affiliations:** Department of Pharmaceutical Biotechnology, Manipal College of Pharmaceutical Sciences, Manipal Academy of Higher Education, Manipal, 576140 India

**Keywords:** Soil isolate, Biopolymer, Bioplastic, Biodegradable, Polyhydroxyalkanoates, Microbial polyesters, Microbiology, Environmental sciences

## Abstract

Population explosion in recent years has driven the environment to overuse nondegradable substances. Microbial polyesters known as polyhydroxyalkanoates (PHAs) are generated and retained as cytoplasmic granules in microorganisms with restricted nutritional availability and can be used to manufacture bioplastics. The current study attempts to screen soil isolates for PHA production and optimize their media parameters. Among all the isolates, 17 were identified and confirmed by Sudan black staining, as they are screening for PHA production and are identified by their colony characteristics. The isolation of the most promising strain, GS-14, was achieved through the sodium hypochlorite method, and subsequent quantification involved establishing a standard curve of crotonic acid. Notably, isolate GS-14 presented the highest yield, which was determined by extrapolating its data onto the standard curve. Characterization of the PHA polymer was subsequently performed, and the results were used to discern its properties. FTIR confirmed characteristic PHA absorption bands, with a prominent C = O stretching peak at 1732 cm⁻¹. LC-MS detected a molecular mass of 641.6 g/mol, indicative of an oligomeric species, while the actual polymer molecular weight is estimated between 5,000 and 20,000 Da. DSC revealed an exothermic peak at 174 °C, allowing the calculation of crystallinity, a key determinant of mechanical properties. Furthermore, the PHA-producing organism was identified as *Bacillus australimaris* through the sequencing of 16 S ribosomal RNA. The media optimization was performed via Minitab software, with statistical analyses employed to interpret the resulting data comprehensively.

**Supplementary Information:**

The online version contains supplementary material available at 10.1038/s41598-025-92146-x.

## Introduction

Plastic manufacturing increased significantly during the 1970s compared to all other materials, resulting in increased pollution and output per the UN environment program. It is predicted that by 2050, the world’s primary plastic output will amount to 1,100 million tonnes if past trends continue^1^. Packaging, which includes single-use plastic goods such as food and beverage containers, uses approximately 36% of all plastics manufactured. Almost 85% of these packing materials are disposed of as unregulated garbage or wind in landfills. Plastic has many applications, from packaging and the food industry to various public health devices, indicating its relevance in modern society^2^. However, poor solid waste management poses a significant threat to the ecological system, affecting the environment and the living system. The use of plastics for various purposes originated around the 18th and 19th centuries, after which there was a sudden surge in their usage over 150 years, rightly referred to as the plastic age^3^.

A flexible approach is needed to address issues associated with landfilling, incineration, and related concerns. This requires a sensible and timely approach to either avoid or minimize the use of these plastics wherever possible, follow better waste management strategies, and include reuse options. In addition to these options, there is an increasing demand for biodegradable plastics, which can solve some of the major global problems faced on a global scale^4^.

Since the degradation process of plastic is very slow and takes approximately 1000 years for synthetic plastic to degrade, biodegradable plastic has attracted much attention on the market because of its degradation time^5^. A potential remedy for plastic pollution involves the use of biodegradable polymers such as “starch, cellulose, chitin, chitosan, lignin, and microbial polyesters” to create bioplastics and diverse biomaterials^6^. Among these, polyhydroxyalkanoates (PHAs) are microbial polyesters that offer significant potential as sustainable and environmentally friendly biomaterials, particularly in bioplastic production^6–9^.

In 1988, the Dutch microbiologist Martinus Willem Beijerinck first discovered polyhydroxyalkanoate (PHA) in microorganisms. In 1925, Lemoigne discovered the first polyhydroxyalkanoate from *Bacillus megaterium*. Its high tensile strength (30–35 MPa) and melting point (175 °C) make it a desirable material. Owing to their promising qualities, such as excellent biodegradability under many conditions, PHAs are becoming more popular among biodegradable polymers^10^. However, owing to the high production cost in industry, the application of these biodegradable plastics has been challenging^11^. This challenge can be mitigated using cost-effective substrates for microorganisms that accumulate PHAs^12^.

Most polyhydroxyalkanoates are synthesized by bacteria. Nutrient deprivation coupled with ample carbon sources stimulates the formation of PHA granules within diverse microbial populations^13–15^. PHAs are hypothesized to serve as traps for carbon and lower equivalents because they aggregate as distinct granules up to 90% of the dry weight of the cell. As their overall fitness is unaffected, it is beneficial for bacteria to store surplus nutrients intracellularly when nutrient sources are unbalanced. The osmotic condition of a cell is not altered by polymerizing soluble precursors into impenetrable molecules, and leakage of these priceless substances from the cell is also stopped^16^.

Recent studies have identified a diverse group of bacteria capable of producing polyhydroxyalkanoates (PHAs), with 31 isolates found across six genera: *Arthrobacter*, *Bacillus*, *Exiguobacterium*, *Halomonas*, *Paracoccus*, and *Rhodobacter*. Among these, *Bacillus* species were the most predominant, a finding consistent with previous research that underscores the genus’s significant role in PHA biosynthesis^17^. As highlighted by Mizuno et al., *Bacillus* bacteria produce class IV PHA synthase, a crucial enzyme for PHA production^18^. However, other genera have also demonstrated considerable potential for PHA synthesis^19,20^. For example, *Exiguobacterium acetylicum* BNL 103 has been shown to produce poly(3-hydroxybutyrate), while species of *Alcaligenes*, *Burkholderia*, *Ralstonia*, *Pseudomonas*, *Paracoccus*, and *Rhodobacter* are known to accumulate various forms of PHAs^21^. Furthermore, *Halomonas* species have emerged as promising candidates for PHA production, with *Halomonas alkalicola* yielding up to 5.9 g of PHA from 100 g of bamboo biomass^22^. These findings highlight the vast potential of various bacterial genera in the sustainable production of biopolymers, emphasizing the need for continued exploration and optimization of microbial strains for PHA production.

Polyhydroxyalkanoates (PHAs) stand out among petrochemical polymers because of their remarkable biodegradability and biocompatibility, which are the defining qualities that distinguish them^23^. These naturally occurring soil-produced polymers undergo degradation when exposed to similar bacteria. Various factors influence PHA biodegradation, including environmental microbial activity, item surface area, temperature, pH, molecular weight, and crystallinity^24,25^. The process begins as microorganisms colonize the plastic surface, secreting enzymes that breakdown the polymer into hydroxy acid monomeric units. Microbes then assimilate these units as carbon sources for their growth and development.

Under aerobic conditions, polymers degrade into carbon dioxide and water, while they yield carbon dioxide and methane under anaerobic conditions. PHA degradation in the environment mainly occurs through enzymatic attack and is considered relatively swift, often involving hydrolytic attack on the ester linkages within the polymers. Notably, PHAs, which are composed mainly of 3-hydroxy acids (and sometimes 4-, 5-, and 6-hydroxy acids), exhibit ester connections that are less susceptible to hydrolysis than those derived from 2-hydroxy acids^26^.

PHAs exhibit high biodegradability in marine environments due to the abundance of microbes that produce extracellular enzymes designed specifically for PHA breakdown, such as P(3HB) depolymerases. These marine microorganisms efficiently metabolize and mineralize the enzymatic breakdown products of PHA, thus completing the carbon cycle not only on land but also in the sea^24,27^.

In addition to their biodegradability, PHAs, notably P(3HB) (poly(3-hydroxybutyrate)) and their breakdown products, such as 3-hydroxy acids, have been found in many organisms. P(3HB) forms hydrophobic ion channel complexes across different life forms in cell membranes. Upon P(3HB) polymer breakdown, R-3-hydroxybutyric acid, a common component of blood, is generated within physiological concentrations and is linked to ketone body synthesis. Medical devices, surgical implants, and sutures made from PHA exhibit excellent biocompatibility, often eliciting no immunological response in the host body. Notably, sterilization procedures have no discernible effect on the standard molecular weight, tensile strength, or other essential attributes of PHA-based materials. Moreover, the surface characteristics of PHA films are conducive to tissue culture cell proliferation and adhesion, making PHA a promising scaffold material for tissue engineering applications. The polymer P(HBco-HV-co-HHx) presents desirable surface roughness and water contact angle, crucial factors supporting cell adhesion and proliferation on PHA surfaces^15,16,28^.

The importance of bioplastics as sustainable alternatives to traditional plastics in addressing environmental pollution is increasing. Microbial biopolymers, promising sources for bioplastic production, remain largely underexplored despite their potential to provide biodegradable materials with reduced ecological impact^29^. The biodiversity across the world, particularly in unique and diverse ecosystems, harbors vast microbial diversity that holds potential for novel biopolymer production. Identifying and isolating bacteria capable of producing these biopolymers is cost-effective and relatively simple, as it can be conducted in a laboratory setting via straightforward techniques. Notably, this paper discusses the most cost-effective and accessible methods for implementing this approach to support sustainable material production and expand our understanding of microbial resources.

This study explores promising avenues for the microbial production of polyhydroxyalkanoates (PHAs) from renewable resources, aiming to replace petroleum-based plastics. Research has focused on isolating PHA-producing bacteria from soil and refining production parameters via the use of agricultural wastes as cost-effective feedstocks. The hypothesis states that soil is a viable source of novel PHA producers, with agricultural wastes effectively yielding high PHA quantities. The study’s outcomes are anticipated to advance the development of sustainable and economical bioplastic manufacturing. To address a key challenge in PHA production, investigations have targeted cost-effective methods to increase yields from natural sources. This study focused on isolating and screening PHA-producing bacteria from diverse soil samples in Manipal, India, with the goal of broadening the pool of high-yield PHA producers. By leveraging various environmental sources, such as soil, novel PHA producers with unique traits can be identified, facilitating large-scale production viability. This study involved the isolation of PHA-producing bacterial strains from the Manipal soil samples, screening for PHA production through Sudan black staining, screening the production parameters via Minitab, and characterization via specialized analytical techniques.

## Materials and methods

### Sample collection

The soil samples were procured from various locations in and around Manipal, Udupi. The longitude and latitude coordinates are as follows: Medicinal Garden, Manipal College of Pharmaceutical Sciences (13.35706°N, 74.78534°E); Tenkabettu garbage soil (13.37729°N, 74.89972°E); Manipal Endpoint Garden (13.36963°N, 74.78608°E); Hoode Beach (13.40764°N, 74.69730°E); and Tenkabettu paddy fields (13.37729°N, 74.89972°E).

### Analytical methods

#### Isolation, screening, quantification, and extraction of PHA-producing bacteria

The superficial soil was removed, and the soil beneath it was collected in sterile tubes following standard soil sampling protocols. Six dilutions (10^− 1^µg/ml, 10^− 2^µg/ml, 10^− 3^µg/ml, 10^− 4^µg/ml,10 ^− 5^µg/ml,10^− 6^µg/ml) of each of the soil samples were made by suspending the required quantity of soil in sterile water followed by serial dilutions. Starch agar served as the nutrient medium for the growth of the organisms. Four different dilutions, i.e., 10^− 1^µg/ml, 10^− 4^µg/ml, 10^− 5^µg/ml, and 10^− 6^µg/ml of all the soil samples were plated individually on starch agar plates. All the plates were incubated at 30 °C for 24 h.

PHA-producing bacteria can be screened by Sudan black staining, as it confirms the presence of intracellular PHA^30^. Sudan black stain was prepared by dissolving 0.3 mg of the powder of Sudan black B in 100 ml (approximately 3.38 oz) of 70% ethanol. The bottle containing the solution was shaken for approximately 5 min and filtered into a fresh bottle. The solution was stable at room temperature. A smear of the bacteria was made on a clean glass slide and heat fixed on the glass slide. The smear was stained with Sudan black and incubated for 10 min. The slides were washed with xylene 3 times for decolorization and counterstained with safranin for 1 min. The slides were washed and air-dried. The cells were observed under a microscope at 100 ×^31,32^. The positive Sudan black-stained isolates were streaked on starch agar plates and incubated for 24 h at 30 °C to obtain pure cultures. After incubation, the colonies were confirmed by Sudan black staining and Gram staining.

The extraction of PHA from the screened organisms was performed via the sodium hypochlorite method. A few colonies were picked with a sterile inoculation loop and transferred to 10 ml of nutrient broth with a composition of D(+)- glucose (1 g/L), sodium chloride (6 g/L), peptone (15 g/L), and yeast extract (3 g/L) supplemented with 1% glucose. The flask containing the inoculum was incubated for 24 h at 30 °C. Five milliliters of broth was added to 50 ml of nutrient broth supplemented with 1% glucose. The above flasks were incubated for 72 h in a shaker incubator at 200 rpm at 30 °C. After 72 h, the culture was centrifuged at 7000 rpm for 15 min. The supernatant was discarded. Then, 5 ml of sterilized water was added, and the mixture was sonicated. After sonication, 5 ml of 20% sodium hypochlorite was added, and the mixture was vortexed and then incubated for 30 min at 37 °C. After incubation, the culture was centrifuged at 7000 rpm for 15 min. A white pellet was obtained and then washed with an acetone: alcohol mixture at a ratio of 1:1 v/v. The obtained pellet was vortexed and dissolved in 10 ml of chloroform. The chloroform was allowed to evaporate by pouring the solution into a glass petri dish and placing it at 28 °C. The obtained powder was quantified after evaporation^33^.

The polymers obtained from the isolates were quantified via the crotonic acid method. A standard plot of crotonic acid was generated. Twenty milligrams of crotonic acid powder was dissolved in 100 ml of 80% sulfuric acid to make a standard stock solution. The standard stock was diluted with concentrated sulfuric acid. Accordingly, twofold dilutions ranging from 50 µg/ml to 0.99 µg/ml were used. The absorbance of the samples was measured at a wavelength of 235 nm via a UV‒visible spectrophotometer. A standard curve of crotonic acid was generated^34^. For the quantification of the polymer, two milligrams of powder were weighed and dissolved in 10 ml of 80% sulfuric acid, which was converted into crotonic acid; the concentration was 0.2 mg/ml. Approximately seven dilutions were made, such as 0.1 mg/ml, 0.05 mg/ml, 0.025 mg/ml, 0.0125 mg/ml, 0.00625 mg/ml, 0.003125 mg/ml, 0.00625 mg/ml, 0.003125 mg/ml, and 0.00156 mg/ml. The seventh dilution was analyzed via UV‒Vis spectrophotometry at 235 nm.

### Characterization of PHA

The characterization of PHA was performed through Fourier transform infrared spectroscopy, liquid chromatography‒mass spectrometry, and nuclear magnetic resonance differential scanning calorimetry.

For Fourier transform infrared spectroscopy analyses, 5 mg of each sample was weighed and dissolved in chloroform. After the evaporation of chloroform, FTIR spectra were recorded using an IR Affinity-1 SHIMADZU^35^.

Liquid-chromatography mass spectrometry was performed via the following procedure: Ten milligrams of the sample was weighed. Methanol and acetonitrile were added, and the samples were analyzed by a Diomex Ultimate 3000 HPLC-hyphenated linear ion trap LTQ XL mass spectrometer (Thermo Fisher).

For nuclear magnetic resonance (NMR), ten milligrams of the sample were weighed and dissolved in CDCL_3_. The samples were analyzed via a BRUKER AscendTM 400 NMR spectrometer.

The thermal properties of the samples were analyzed via differential scanning calorimetry via a Shimadzu thermal analyzer over a temperature range of 20 °C to 500 °C. Approximately 5 mg of each sample was utilized for the analysis. The melting temperature (Tm) was determined from the heat runs. The melting enthalpy of the sample (ΔHm) was compared with the melting enthalpy of 100% crystalline PHA (ΔH0m) to determine the degree of crystallinity (Xc).

### Identification of the organism

The samples were outsourced to Himedia to identify the organisms, where the following process identified the isolate.

Each sample received at Hi-Gx360^®^ is anonymized to an HM Code for unbiased processing, uniformity, and easy tracking. The bacterial 16S rRNA gene served as the target for amplification via the universal primers 16S27F “(5’-CCA GAG TTT GAT CMT GGC TCA G-3’)” and 16S1492R “(5’-TAC GGY TAC CTT GTT ACG ACT T-3’)”^36^. The amplified PCR products were purified via salt precipitation. Agarose gel electrophoresis was subsequently conducted to assess the quality of the PCR amplicons and postpurification PCR products. The purified amplicons were subjected to cycle sequencing via BDT v3.1 chemistry and sequenced on an ABI 3500XL Genetic Analyzer. Additional internal primers were employed to obtain near-full-length sequences, ensuring that high-quality base reads covered the target region. The resulting ab1 trace files underwent manual curation, conversion into fasta read files, assembly into contiguous sequences, and exportation into FASTA files. The consensus sequence was subjected to a database search against the SILVA database v138^37^ via the BLAST tool^38^.

For the phylogenetic analysis, up to 10 closest-neighbor sequences belonging to different taxa from the top 1000 hits with the highest similarity in the search results were retrieved from the database and aligned via the MUSCLE aligner^39^. The multiple sequence alignment was manually inspected and used to produce a consensus phylogram via a maximum likelihood/neighbor-joining algorithm with 1000 iterations via MEGA11 (Molecular Evolutionary Genetic Analysis, version 11) software^40^. The goal of a phylogenetic tree is to identify its topology, which, at the points of its branches, most likely contributes to the species’ observable traits.

### Screening of media components and parameters for PHA production

To optimize PHA production via various media components and analyze different process parameters, the Plackett–Burman design (PBD) was employed. PBD is typically utilized to screen different factors in an experiment. This study aimed to analyze the optimal production of PHA by *Bacillus australimaris* with starch as a significant carbon source.

Interactions between components were considered insignificant. A complete factorial design would be needed to meet the experimental design requirement where any combination of levels for every pair of components appears simultaneously throughout all experimental runs. However, the aim was to identify smaller designs. Nine factors were selected based on the results obtained during the one factor at a time (OFAT) experimental runs as well as from the literature reviews, with high and low levels coded as + 1 and − 1, respectively, at a 95% relative significance level in Minitab to conduct the experiments. The experiments were performed on the basis of the runs obtained from the software, and PHA production was analyzed as the primary response variable. The various experimental parameters used for PHA synthesis via the PBD are listed in Table 1.

**Table 1:** Different levels of experimental factors selected for the PHA via PBD.

Sl.no	Components (Factors)	Low level (-1) g/l	High level (+ 1) g/l
1.	Di sodium hydrogen phosphate (Na_2_HPO_4_)	0.5	5.0
2.	Potassium dihydrogen phosphate (KH_2_PO_4_)	1.5	15.0
3.	Ammonium sulphate ((NH_4_)_2_ SO_4_)	1.5	15.0
4.	Magnesium sulphate (MgSO_4_)	0.2	2.0
5.	Starch	20	40
6.	Temperature	30	32
7.	pH	7.0	7.5
8.	RPM	120	200
9.	Incubation time	72	80

PHA production was conducted by cotton-plugging 250 ml and 100 ml Erlenmeyer flasks filled with 50 ml of the production medium, as outlined in the supplementary data. The medium composition included Na_2_HPO_4,_ KH_2_PO_4_, (NaH_4_)_2_ SO_4,_ MgSO_4_, and starch as the carbon source. Potato starch was used as the starch source. All flasks were autoclaved for sterilization and cooled to room temperature. The pH was adjusted to seven before the organisms were inoculated. A 5% inoculum of the PHA-producing organism was added to all flasks, followed by incubation at various temperatures and RPMs corresponding to low and high levels.

## Results and discussion

### Isolation and screening of PHA-producing bacteria

**Fig. 1:**
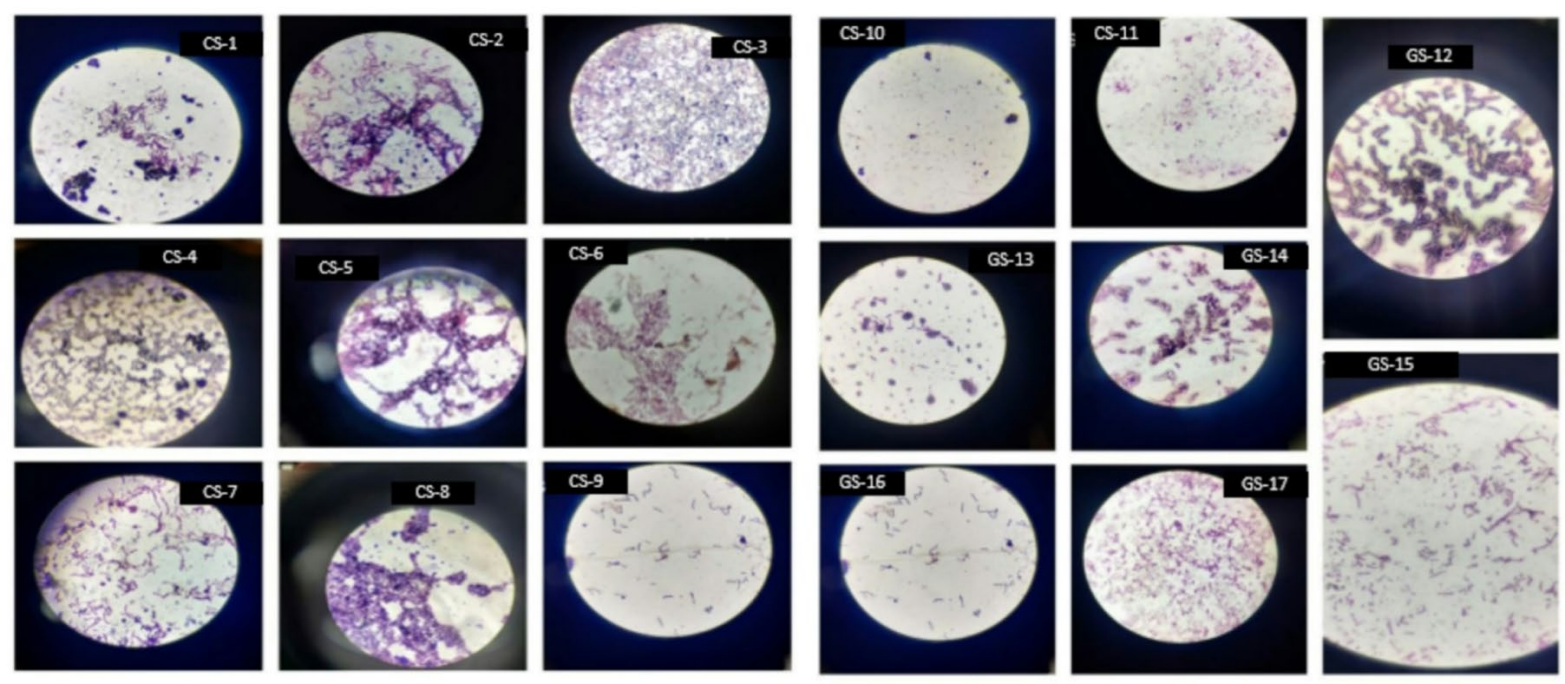
Sudan Black staining of isolates 1 to 17, highlighting the presence of lipid inclusions within the cells. The stained lipid droplets appear as dark or black regions, indicating lipid accumulation, which is characteristic of these isolates.

**Fig. 2:**
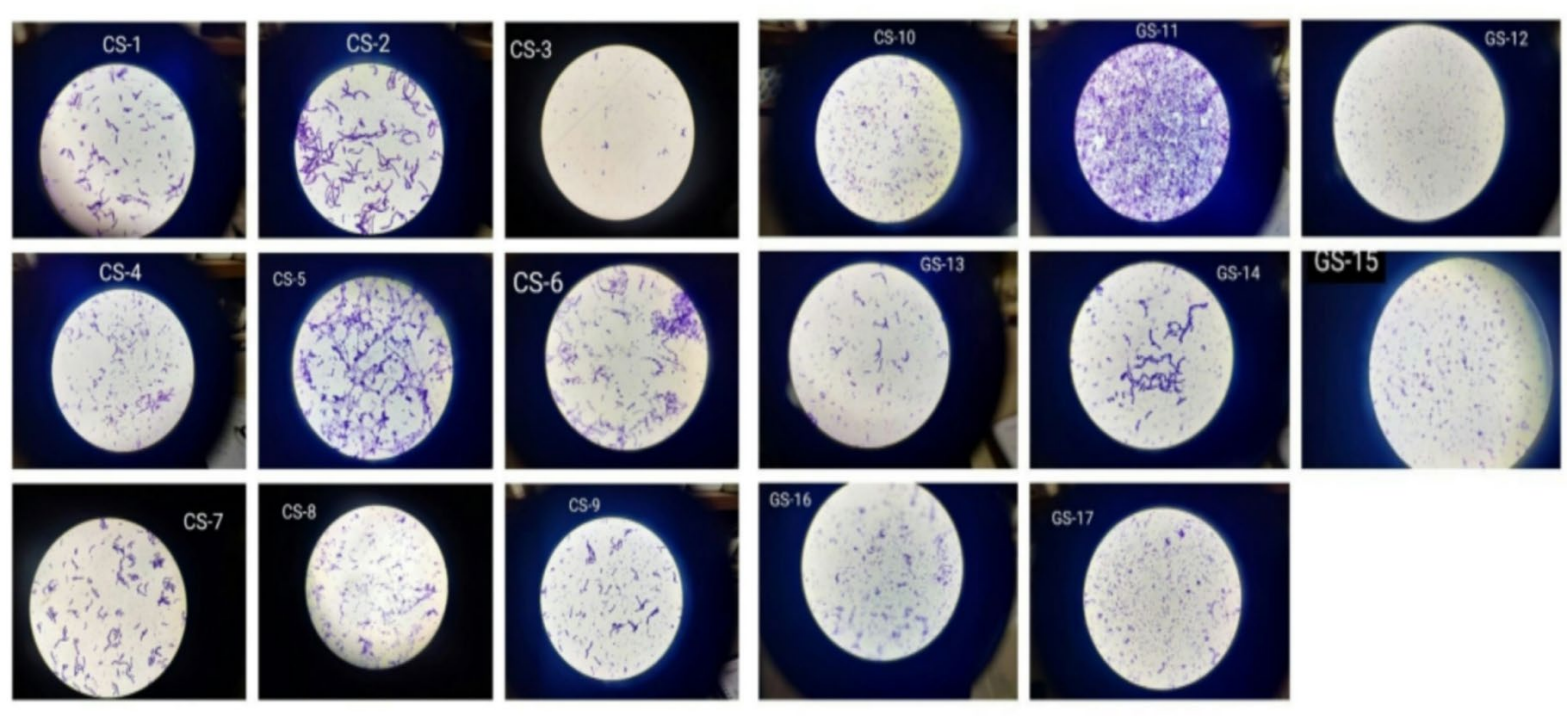
Gram staining results for isolates 1–17. The figure depicts the Gram staining results for 17 bacterial isolates, which were differentiated on the basis of their Gram-positive or Gram-negative characteristics. A purple or violet color is characteristic of Gram-positive bacteria, whereas Gram-negative bacteria appear pink or red under a microscope.

Primary and secondary screenings were conducted to identify potential PHA-producing bacterial isolates. Soil samples were serially diluted and plated, yielding dense growth on undiluted plates and distinct colonies on diluted plates. Sudan Black staining was employed for initial screening, where isolates exhibiting intense black pigmentation, indicative of lipid accumulation, were selected for further analysis. Isolates with weaker pigmentation were excluded from subsequent studies. Secondary screening was performed using pure cultures grown on starch agar to isolate single colonies. These colonies underwent Sudan Black staining to confirm PHA production (Fig. 1). All 17 selected isolates were identified as Gram-positive bacteria (Fig. 2).

The collected isolates were subjected to microscopic examination for color, shape, motility, and Gram staining. Macroscopic characteristics such as size, shape, texture, color, growth pattern, margin opacity, and pigment production were also analyzed. The detailed results are provided in Tables 2 and 3.

**Table 2:** Microscopic characterization of the soil isolates.

Isolate no.	Color	Shape	Motility	Gram-Staining
CS-1	Purple	Rod	Yes	Positive
CS-2	Purple	Rod	Yes	Positive
CS-3	Purple	Rod	Yes	Positive
CS-4	Purple	Rod	Yes	Positive
CS-5	Purple	Rod	Yes	Positive
CS-6	Purple	Rod	Yes	Positive
CS-7	Purple	Rod	Yes	Positive
CS-8	Purple	Rod	Yes	Positive
CS-9	Purple	Rod	Yes	Positive
CS-10	Purple	Rod	Yes	Positive
GS-11	Purple	Rod	Yes	Positive
GS-12	Purple	Rod	Yes	Positive
GS-13	Purple	Rod	Yes	Positive
GS-14	Purple	Rod	Yes	Positive
GS-15	Purple	Rod	Yes	Positive
GS-16	Purple	Rod	Yes	Positive
GS-17	Purple	Rod	Yes	Positive

**Table 3:** Macroscopic characteristics of the soil isolates.

No	Size	Shape	Texture	Color	Growth	Margin	Opacity	Pigment
CS-1	Moderate	Circular	Smooth	Creamy white	Abundant	Even	Opaque	No
CS-2	Moderate	Circular	Smooth	Creamy White	Abundant	Even	Opaque	No
CS-3	Moderate	Irregular	Smooth	Yellow	Abundant	Wavy	Opaque	No
CS-4	Large	Irregular	Rough	White	Spreading	Wavy	Opaque	No
CS-5	Small	Punctiform	Smooth	White	Limited	Wavy	Translucent	No
CS-6	Large	Irregular	Smooth	White	Abundant	Wavy	Opaque	No
CS-7	Moderate	Circular	Smooth	Yellow	Abundant	Wavy	Opaque	No
CS-8	Small	Punctiform	Smooth	Yellow	Abundant	Wavy	Opaque	No
CS-9	Moderate	Circular	Smooth	Yellow	Abundant	Even	Opaque	No
CS-10	Moderate	Irregular	Rough	White	Spreading	Wavy	Opaque	No
GS-11	Large	Irregular	Rough	Creamy white	Spreading	Wavy	Opaque	No
GS-12	Small	Punctiform	Smooth	Creamy white	Limited	Wavy	Opaque	No
GS-13	Small	Punctiform	Smooth	Yellow	Abundant	Even	Opaque	No
GS-14	Moderate	Circular	Smooth	Yellow	Abundant	Even	Opaque	No
GS-15	Small	Punctiform	Smooth	Creamy white	Abundant	Even	Translucent	No
GS-16	Small	Punctiform	Smooth	White	Spreading	Wavy	Translucent	No

Sudan Black B staining was utilized to detect lipid inclusions, such as neutral fats, phospholipids, and sterols, due to its high solubility in triglycerides. Positive staining was characterized by the presence of black pigmentation, confirming the production of PHA. All 17 isolates demonstrated positive results, establishing their capacity for PHA production. These findings underscore the potential of the selected isolates for further investigation in PHA biosynthesis.

### Extraction of polyhydroxyalkanoate

**Fig. 3:**
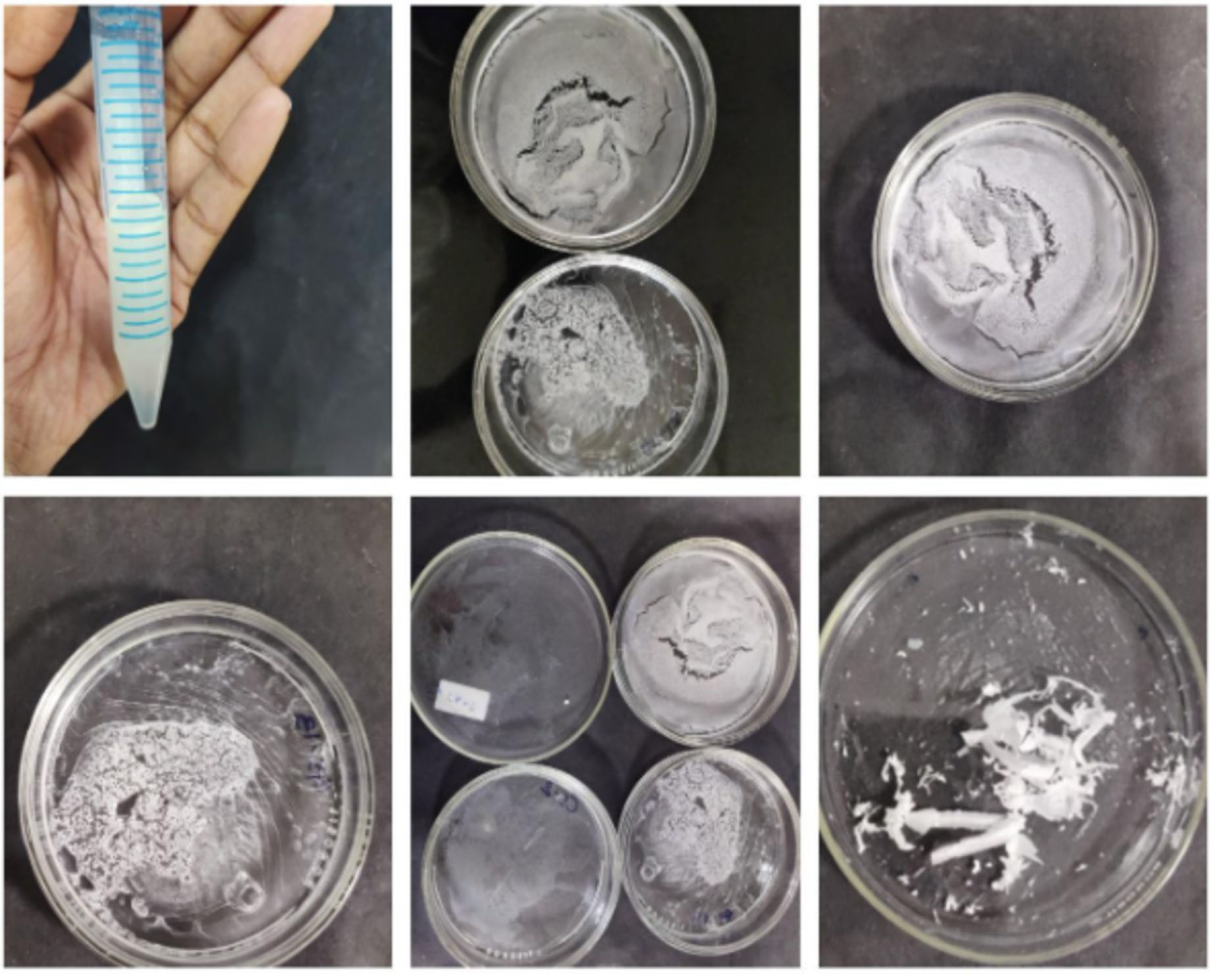
Extraction of polyhydroxyalkanoate.

The polyhydroxyalkanoate from seventeen isolates, confirmed via positive Sudan black staining, were extracted via sodium hypochlorite. Among these, five isolates presented high yields of polyhydroxyalkanoate (PHA). Notably, isolate GS-14 presented the highest yield and was further quantified. Figure 3 shows the extraction of PHA.

### Quantification of PHA

**Fig. 4:**
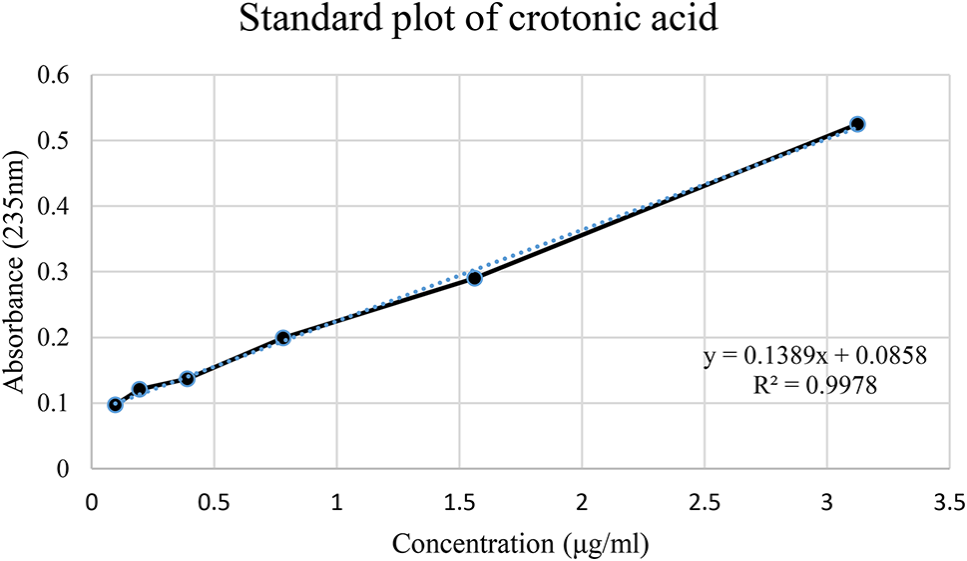
Extrapolation of the Most Promising Isolate (GS-14) via the Standard Curve of Crotonic Acid. This figure shows the quantification of crotonic acid produced by the most promising isolate, GS-14. The values were extrapolated via a standard curve for crotonic acid, enabling precise estimation of its production levels.

A standard curve of crotonic acid was constructed to correlate the absorbance with the concentration via a UV‒Vis spectrophotometer. The absorbance (at 235 nm) of the PHA produced by various isolates was quantified, measured, and extrapolated to a standard curve (Fig. 4).

### Characterization of PHA

#### Fourier transform infrared spectroscopy

**Fig. 5:**
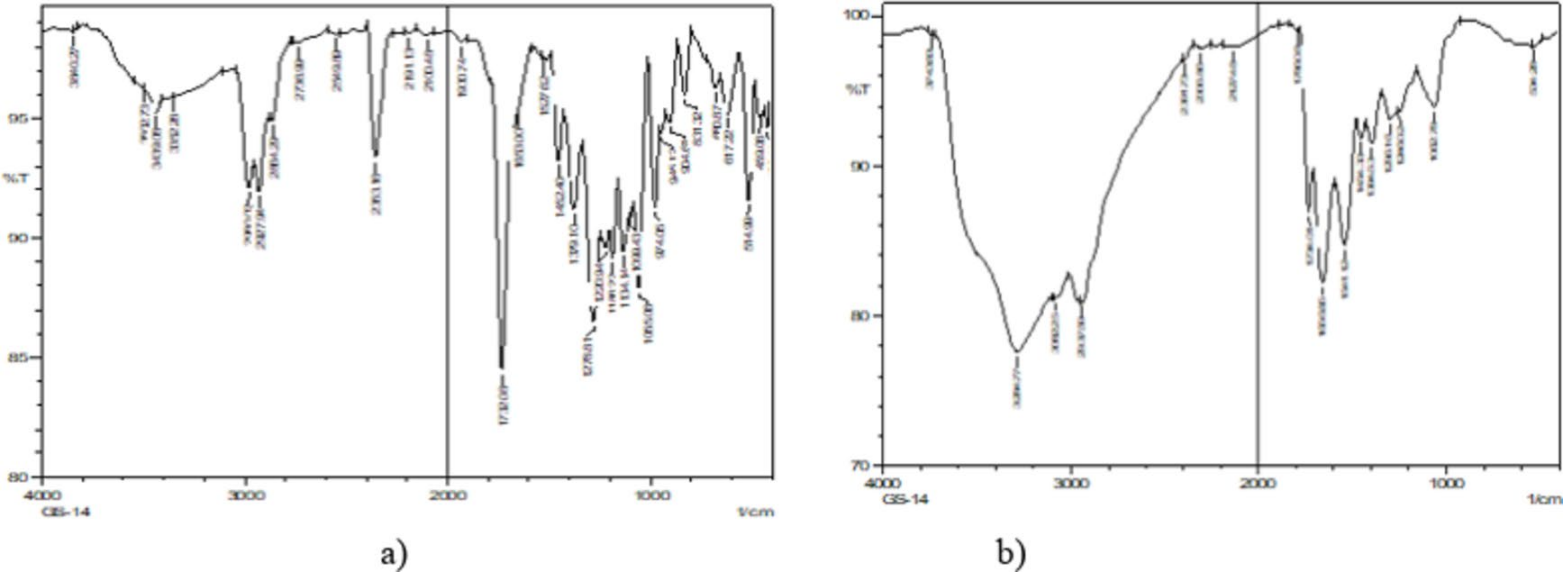
(**a**) Fourier transform spectroscopy of standard PHA, (**b**) FTIR of the PHA from strain GS-14.

The Fourier transform infrared spectroscopy results revealed nearly identical structures of the standard PHA (Fig. 5a) and the biopolymer Bacillus australimaris (GS-14) produced (Fig. 5b). Characteristic bands indicative of PHA were observed in the sample designated GS-14. The most prominent band, located at 1732 cm^− 1^, corresponds to the stretching of the C = O bond, whereas a series of intense bands within the range of 1–1300 cm^− 1^ correspond to the C-O bond of the ester group. Additionally, the band at 1457 cm^− 1^ corresponds to asymmetrical deformation. Minor bands of lesser significance were observed at 3440 cm^− 1^. The FTIR spectra of the biopolymers produced from various isolates are provided in the supplementary data.

#### Liquid chromatography-mass spectrometry

The mass spectrometric analysis of the extracted polymer was performed using Ion Trap Mass Spectrometry (ITMS) with Atmospheric Pressure Chemical Ionization (APCI) in positive mode. The obtained spectra provide insights into the polymer’s molecular composition and oligomeric distribution.

The upper spectrum (GS-14 #70–121, RT: 0.34–0.56 min) exhibited a base peak at m/z 255.28, with additional prominent peaks at m/z 157.12, 219.20, and 283.35. Signal intensity decreased significantly beyond m/z 500, with only a few low-abundance peaks extending toward m/z 2000.

The lower spectrum (GS-14 #76–113, RT: 0.37–0.54 min) demonstrated a broader range of peaks, suggesting a polymeric distribution. Distinct peaks at m/z 143.03, 181.00, 209.20, 277.22, 313.38, 383.44, 439.47, 495.54, 551.64, 607.75, 663.59, and 783.93 were observed. Notably, the regularly spaced peaks, approximately 60 Da apart, indicate the presence of a repeating monomeric unit, a hallmark feature of polyhydroxyalkanoates (PHAs).

The spectral data strongly suggest that the analyzed polymer is a PHA, likely poly(3-hydroxybutyrate) (PHB) or a related copolymer. The detection of a repeating unit of approximately 60 Da is consistent with 3-hydroxybutyrate (3HB) monomers commonly found in PHAs. Additionally, peaks extending beyond m/z 1800 + indicate the presence of a high-molecular-weight polymer. The base peak at m/z 255.28 suggests a low-molecular-weight oligomer or a characteristic fragmentation product, while peaks at m/z 495, 551, 607, and 663 further support PHA oligomers with varying chain lengths.

**Fig. 6:**
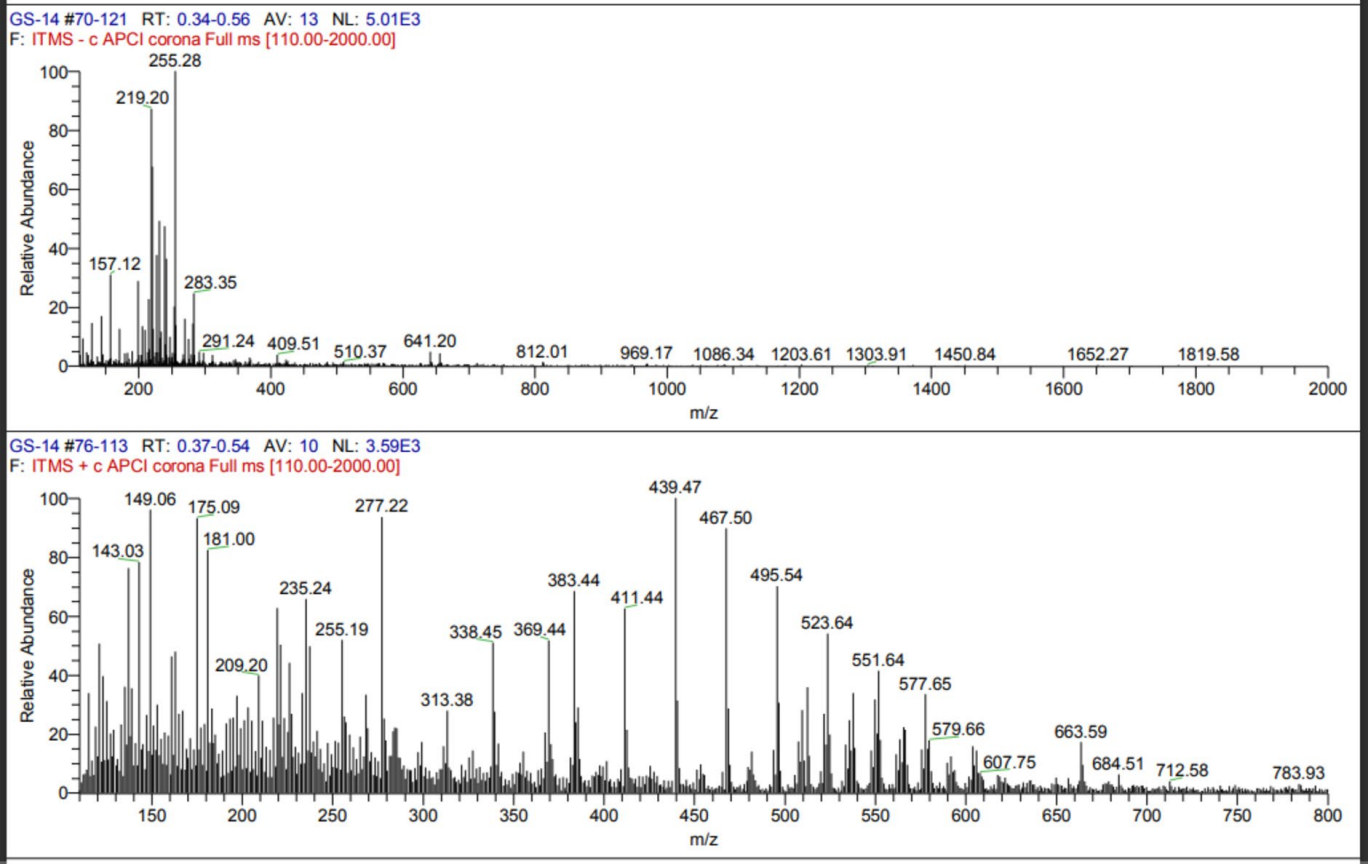
The mass spectrum of standard PHA.

While the exact molecular weight of the polymer could not be determined solely from this mass spectrometric analysis, the detected mass distribution and the presence of repeating units strongly support the hypothesis that the isolated polymer is a PHA with an estimated molecular weight in the range of 5,000–20,000 Da, consistent with literature reports for bacterial PHAs. The mass spectrum of the standard PHA is depicted in Fig. 6.

#### Nuclear magnetic resonance

**Fig. 7:**
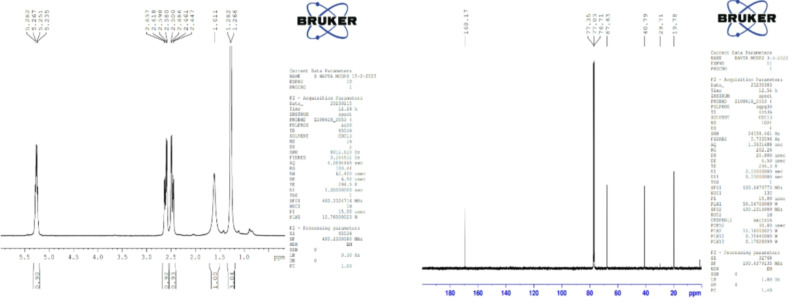
(**a**) 1 H NMR spectrum of standard PHA. (**b**) 13 CNMR spectrum of the standard PHA.

The structure of PHA, as demonstrated in Fig. 3, was confirmed by a 1 H NMR investigation (Fig. 7a). PHA spectrum analysis revealed that the standard had two monomeric units. For -CH3, -CH2, and OH, resonances were detected at 1.282, 2.500, and 5.282 ppm, respectively. Figure 7b presents the 13CNMR spectra. The spectra show that the peaks at 19.78, 40.79, 67.63, and 169.15 ppm correlate to the carbon atom types found in the PHA structure, namely, CH3, CH, CH2, and C = O, respectively.

#### Differential scanning calorimetry

**Fig. 8:**
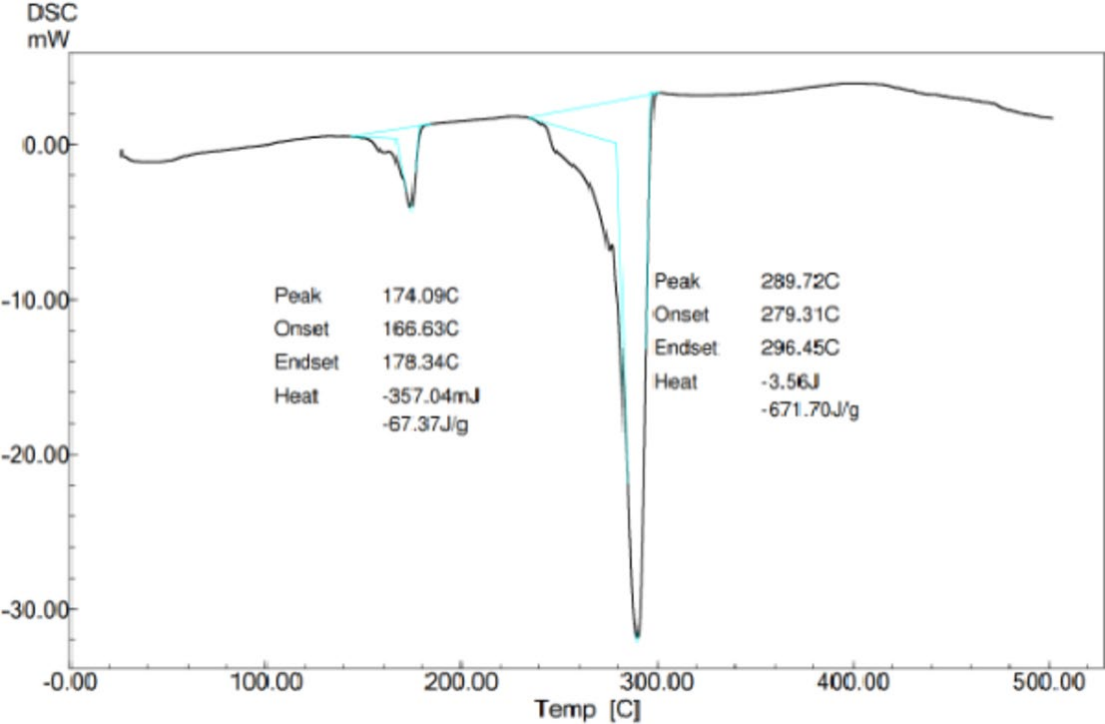
Differential scanning calorimetry of the standard PHA.

The thermal behavior of the standard PHA was investigated via differential scanning calorimetry, and the results are shown in Fig. 8. The peak at 174 °C is exothermic, and the melting enthalpy allows the degree of crystallinity (Xc), which is the most critical characteristic of a polymer since it determines the material’s mechanical properties, to be calculated.

The combined spectral analyses from Mass Spectrometry (MS), Fourier Transform Infrared Spectroscopy (FTIR), and Carbon-13 Nuclear Magnetic Resonance (13 C NMR) strongly suggest that the polymer produced by the bacterial isolate is a polyhydroxyalkanoate (PHA), most likely poly(3-hydroxybutyrate) (PHB) or a closely related PHA copolymer. Based on these findings, the polymer isolated from the bacterial strain GS-14 is conclusively identified as a PHA, most likely PHB or a closely related copolymer. These results provide strong evidence that the microorganism has the potential for efficient PHA biosynthesis, supporting its applicability for biopolymer production.

### Identification of the isolate

**Fig. 9:**
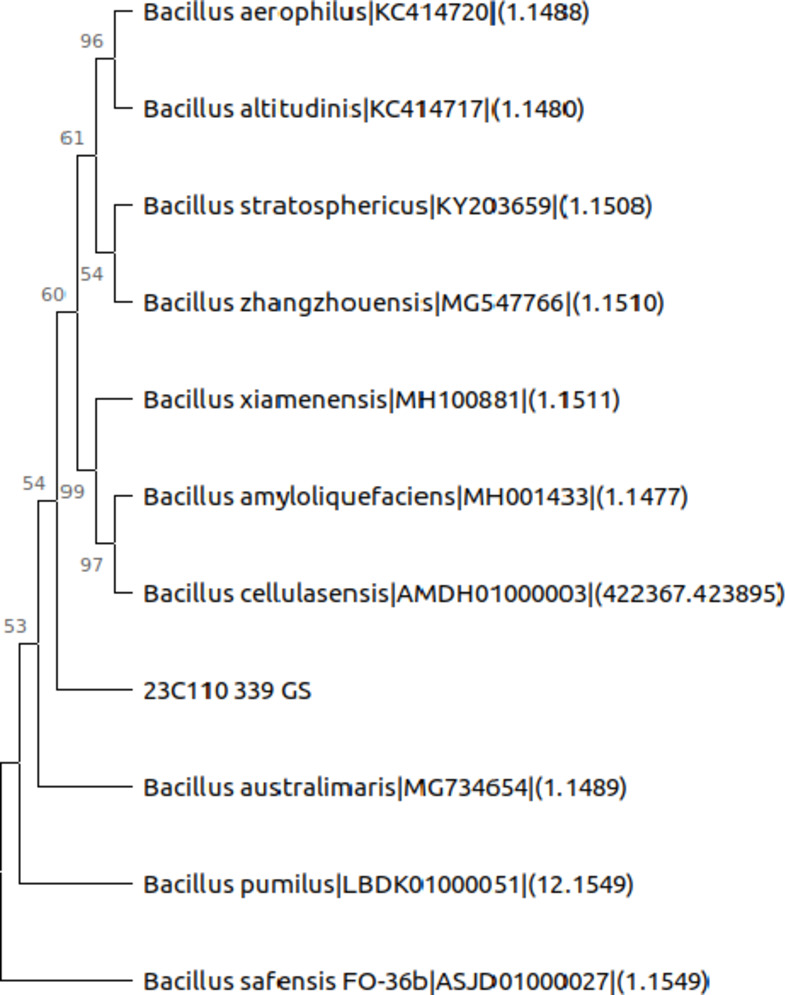
Phylogenetic tree of closely related microorganisms.

The sequencing and phylogenetic analysis of the sequence revealed that the isolated organism was *Bacillus australimaris* (Fig. 9). The sequences are deposited in the NCBI repository, and an accession number was assigned (SUB14851199 23C110_339_GSPQ569061). The sequence electropherogram is given in the supplementary data.

A phylogenetic tree graphically represents the evolutionary relationships among groups of organisms. This tree comprises branches, each representing a taxon compared within the tree. Branches originating from a common point, termed a node, depict taxa that have evolved from their common ancestor. Trees can be rooted or unrooted, with a common root representing the most ancestral taxon from which all taxa within the tree likely evolved.

The length of branches is crucial for calculating the evolutionary distance between two organisms. Thus, the tree’s topology is more relevant within the context of branch lengths rather than their vertical arrangement. The horizontal lines denote branches and signify evolutionary lineages that changed over time. The longer the branch is in the horizontal dimension, the more significant the genetic change. The numeric values at the nodes represent bootstrap values as percentages, indicating the frequency at which branches were replicated with the same arrangement in the iterations carried out. A higher bootstrap value reflects greater confidence in the branch.

### Screening and optimization of media components

Software-based experimental trials were designed via Minitab 17, with experiments conducted sequentially. The primary aim of this screening study was to identify the parameters that most significantly affect PHA production. Responses in terms of biomass and PHA yield were measured in grams per liter, assessing the relative significance of five factors: Na_2_HPO_4_, KH_2_PO_4_, (NH_4_)_2_SO_4_, MgSO_4_·7H_2_O, and starch concentration at two levels (low and high). After all low-level and high-level values were incorporated into Minitab, the software indicated that 12 runs and subsequent runs yielded responses for PHA and biomass. Statistical analysis of the Plackett–Burman design on biomass yield and PHA yield with nine fermentative parameters is given in Tables 4 and 5. Statistical analysis and Pareto charts of Plackett–Burman for PHA production (g/L) and biomass yield are provided in Table 6.

**Table 4:** Statistical analysis of the effects of the Plackett–Burman design on biomass yield with nine fermentation parameters.

Source	DF	Adj SS	Adj MS	F Value	*P*- F Value
Model	9	0.234447	0.026050	19.64	0.049
Linear	9	0.234447	0.026050	19.64	0.049
Na_2_HPO_4_	1	0.003929	0.003929	2.96	0.227
KH_2_PO_4_	1	0.000108	0.000108	0.08	0.802
(NH_4_)_2_SO_4_	1	0.025571	0.025571	19.28	0.048
MgSO_4_	1	0.010190	0.010190	7.68	0.109
Starch	1	0.000386	0.000386	0.29	0.644
Temperature	1	0.001370	0.001370	1.03	0.416
pH	1	0.021171	0.021171	15.96	0.057
Rpm	1	0.006349	0.006349	4.76	0.160
Incubation time	1	0.002020	0.002020	1.52	0.343
Error	2	0.002653	0.001326		
total	11	0.237100			

**Table 5:** Statistical analysis of the effects of the Plackett–Burman design on the PHA yield with nine fermentation parameters.

Source	DF	Adj SS	Adj MS	F Value	*P*- F Value
Model	9	2.49939	0.27771	0.46	0.829
Linear	9	2.49939	0.27771	0.46	0.829
Na_2_HPO_4_	1	0.02205	0.02205	0.04	0.866
KH_2_PO_4_	1	1.5443	1.5443	1.75	0.316
(NH_4_)_2_SO_4_	1	1.30024	1.30024	2.16	0.279
MgSO_4_	1	1.31842	1.31842	2.19	0.277
Starch	1	0.06927	0.06927	0.12	0.767
Temperature	1	0.55945	0.55945	0.93	0.436
pH	1	0.11985	0.11985	0.20	0.699
Rpm	1	0.21062	0.21062	0.35	0.614
Incubation time	1	0.07913	0.07913	0.13	0.751
Error	2	1.20199	0.60099		
total	11	3.70137			

**Table 6:** The 12 runs suggested by the software.

Sl.no		1	2	3	4	5	6	7	8	9	10	11	12
Std order		1	7	6	3	9	11	5	10	2	4	12	8
Run order		1	2	3	4	5	6	7	8	9	10	11	12
Pt Type		1	1	1	1	1	1	1	1	1	1	1	1
Blocks		1	1	1	1	1	1	1	1	1	1	1	1
Na_2_HPO_4_		5.0	0.5	5.0	0.5	0.5	0.5	5.0	5.0	5.0	5.0	5.0	0.5
KH_2_PO_4_		1.5	15.0	15.0	15.0	1.5	15.0	15.0	1.5	15.0	1.5	15.0	1.5
(NH_4_)_2_SO_4_		15.0	1.5	15.0	15.0	1.5	1.5	1.5	1.5	1.5	15.0	1.5	15.0
MgSO_4_ ·7H_2_O		0.2	0.2	0.2	0.2	2.0	0.2	2.0	0.2	2.0	0.2	0.2	0.2
Starch		20	40	20	40	40	20	40	20	20	40	40	20
Temp		30	32	32	30	32	32	30	32	30	32	30	30
pH		7.5	7.5	7.0	7.0	7.0	7.5	7.5	7.5	7.0	7.0	7.0	7.5
Rpm		200	120	200	120	200	200	120	120	200	120	120	200
Incubation Time		80	80	72	80	80	72	72	80	80	72	72	72
RESPONSES	PHA	1.045	0.634	1.779	1.844	1.167	1.066	2.175	1.182	1.153	0.858	0.404	0.227
	Biomass	0.94	1.27	1.00	1.22	1.37	1.07	1.29	1.16	1.34	1.15	1.30	0.99

**Fig. 10:**
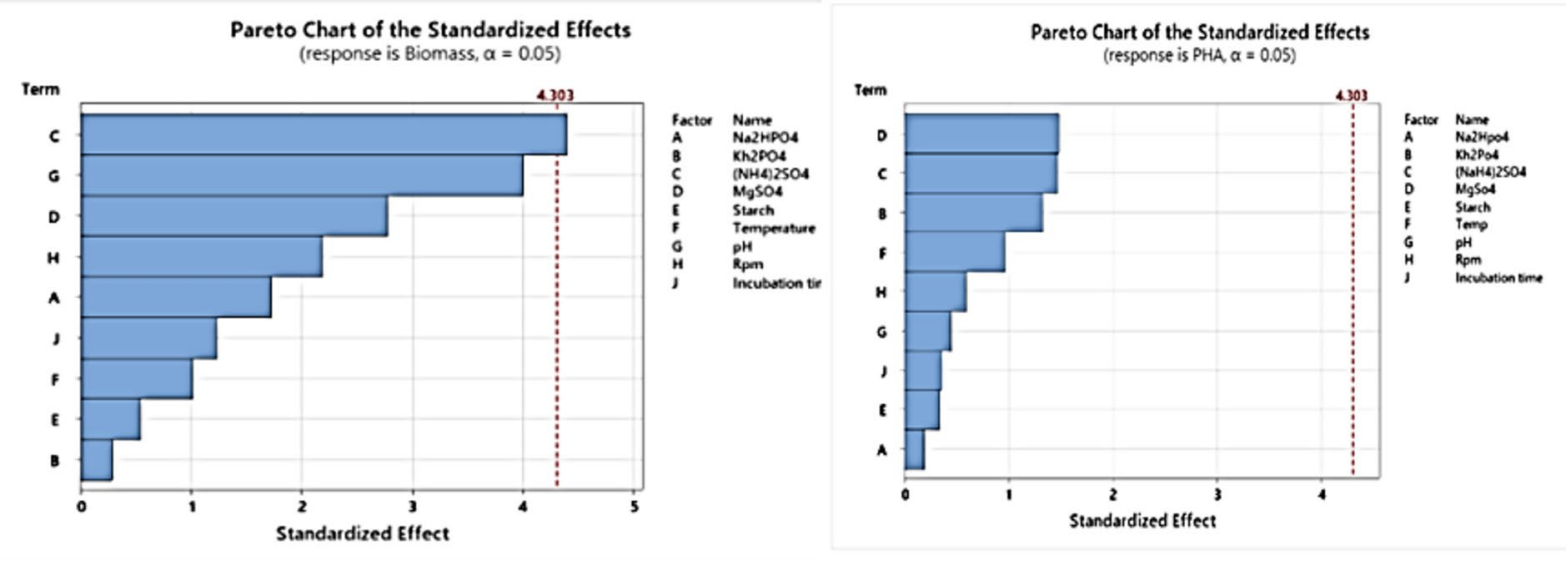
The figure illustrates two Pareto charts of standardized effects, representing the influence of various factors on biomass production (left) and PHA (polyhydroxyalkanoate) production (right). The red dashed line indicates the threshold for statistical significance (standardized effect = 4.303) at an alpha level of 0.05. For biomass production, (NH_4_)_2_SO_4_ (C) was identified as the most significant factor, followed by pH (G) and starch (D), which also surpassed the significance threshold. Conversely, for PHA production, starch (D) had the greatest influence, followed by (NH_4_)_2_SO_4_ (C) and KH_2_PO_4_ (B), all of which were statistically significant. Factors below the threshold, such as NH_4__2_SO_4_ (A), temperature (F), and incubation time (J), have minimal effects on both responses. These results highlight the critical factors for optimizing experimental conditions to increase biomass and PHA production.

Pareto charts (Fig. 10) revealed that the concentrations of three factors, Na_2_HPO_4_, KH_2_PO_4_, and (NH_4_)_2_SO_4,_ positively influenced the PHA yield. Factors with independent variables above 95% were significantly influential on PHA yield. The objective of the screening experiment was to assess the factors that significantly influence PHA production. Plackett–Burman studies were conducted before screening highly influential parameters in production, providing insights into the potential factors affecting production. The factors chosen for the experiments were closely associated with production during previous shake flask cultivations.

The Pareto chart offered a clear visualization of the standardized effect of each factor on production. During the biomass increase, (NH_4_)_2_SO_4_ was found to play a more significant role, followed by pH and starch. The results indicated that the concentration of starch, followed by KH_2_PO_4_ and (NH_4_)_2_SO_4_, notably impacted overall PHA production during growth. Na₂HPO₄, temperature, and incubation time were found to have an insignificant role in PHA production, as was biomass, supporting the notion that a limited nitrogen source can increase PHA production.

**Fig. 11:**
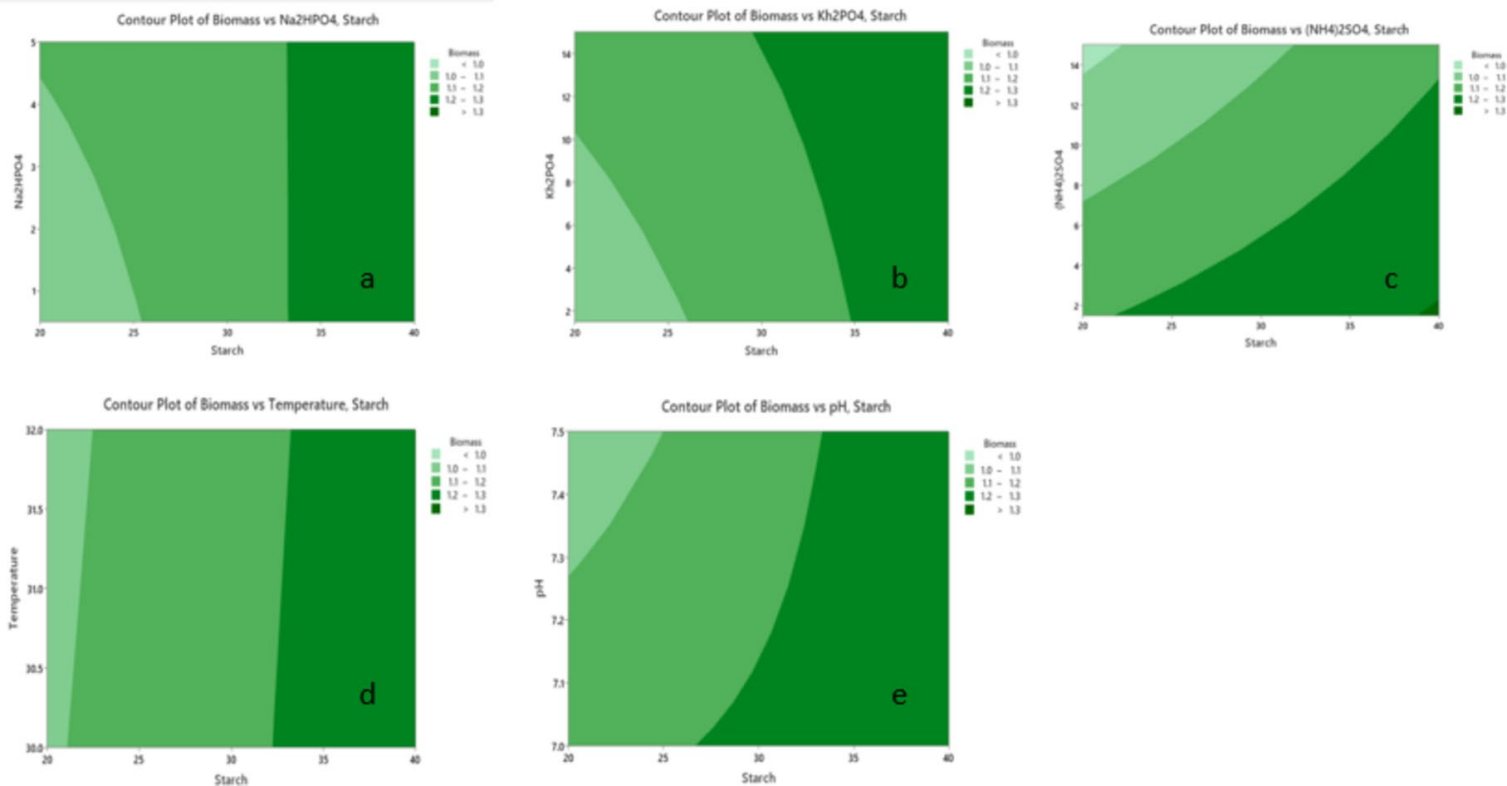
Contour plots showing the effects of starch in combination with NH_4__2_SO_4_ (**a**), KH_2_PO_4_ (**b**), (NH_4_)_2_SO_4_ (**c**), temperature (**d**), and pH (**e**) on biomass production. Biomass levels, represented by a light-to-dark green gradient, indicate that starch is a critical factor in all plots, with higher concentrations consistently increasing biomass production. In the case of (NH_4_)_2_SO_4_ (**c**), a strong interaction with starch is observed, leading to maximum biomass production at elevated levels of both factors. Compared with starch, NH_4__2_SO_4_ (**a**) and KH_2_PO_4_ (**b**) have moderate effects, whereas temperature (**d**) has minimal influence. In contrast, pH (**e**) has a significant interactive effect, with optimal biomass production occurring at relatively high starch concentrations and slightly alkaline conditions. These results highlight starch as the dominant factor influencing biomass, with NH_4__2_SO_4_ and pH also playing important roles.

**Fig. 12:**
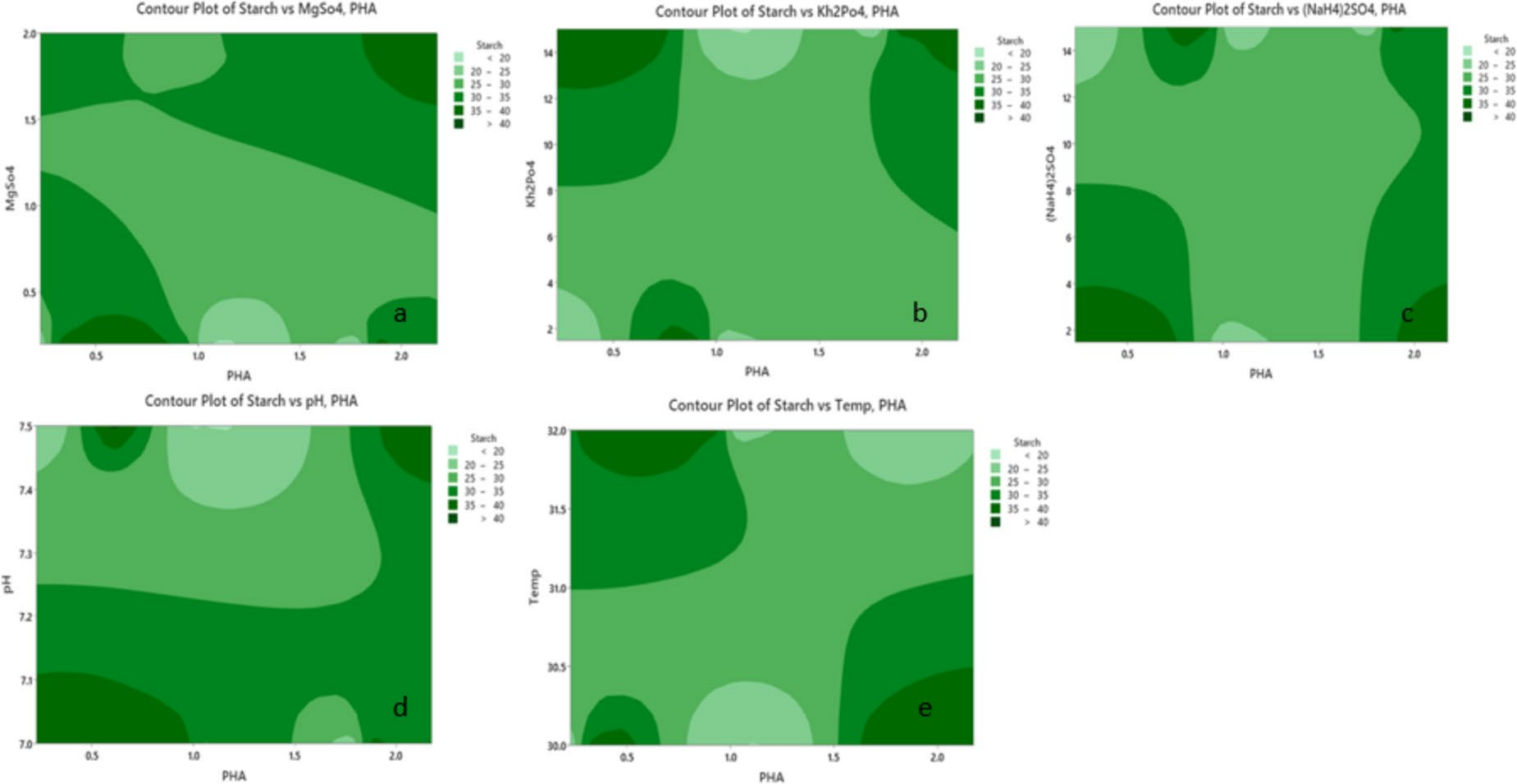
Contour plots illustrating the combined effects of starch with MgSO_4_ (**a**), KH_2_PO_4_ (**b**), (NH_4_)_2_SO_4_ (**c**), pH (**d**), and temperature (**e**) on PHA (polyhydroxyalkanoate) production. The color gradient, ranging from light to dark green, represents varying starch concentrations and their corresponding influence on PHA yield. In all plots, starch significantly impacted PHA production, with higher concentrations generally associated with increased PHA levels. Among the interacting factors, (NH_4_)_2_SO_4_ (**c**) has a notable synergistic effect with starch, maximizing PHA production at elevated levels of both variables. MgSO_4_ (**a**) and KH_2_PO_4_ (**b**) exhibit moderate interactions, whereas temperature (**e**) and pH (**d**) have more nuanced effects, with optimal PHA production occurring within specific ranges of these variables. These results emphasize starch as a critical determinant of PHA production, with (NH_4_)_2_SO_4_ playing an essential complementary role.

Typically, 2D contour plots (Figs. 11 and 12) are utilized as visual tools to examine regression equations and explore the interaction between independent and dependent variables. These plots depict two response factors while holding other variables constant, with the shape of the plot often indicating the extent of interaction. To study the significant impacts of the three factors on biomass and PHA accumulation, 2D plots were used to analyze the interaction effects successfully. The plots between the five variables KH_2_PO_4,_ Na_2_HPO_4,_ (NH_4_)_2_SO_4_, pH, and temperature revealed a significant interaction between these factors and starch in enhancing biomass accumulation. Additionally, the contour plot illustrating PHA production with respect to the KH_2_PO_4_ and Na_2_HPO_4_ concentrations indicated that high concentrations of both factors were directly correlated with increased PHA production.

## Discussion

The isolation and screening of polyhydroxyalkanoate (PHA)-producing bacteria were conducted to identify efficient candidates for high-yield PHA production, which is essential for bioplastic development. The soil samples collected from various locations were processed, and the isolates were subjected to initial screening via Sudan black staining, which revealed lipid-accumulating cells. Only those showing intense black pigments were selected for further testing, ensuring that only robust PHA-producing strains were advanced. Secondary screening on starch agar further confirmed PHA production in selected isolates. This process yielded 17 gram-positive bacteria, which were evaluated for microscopic and macroscopic characteristics, including cell shape, color, motility, colony texture, and pigmentation. Detailed observations provided insights into each isolate’s morphological properties, laying the foundation for precise characterization and selection.

Sudan black staining was performed again on pure colonies to confirm the presence of PHA, which demonstrated successful PHA production across all the isolates. Extraction was then performed via sodium hypochlorite, with five strains showing promisingly high yields, and GS-14, which emerged as the top producer, was isolated. This strain was selected for further analysis and quantified against a standard curve of crotonic acid via a UV‒Vis spectrophotometer, confirming the efficiency of this isolate for PHA production. The biochemical characterization of the extracted PHA involved Fourier transform infrared spectroscopy (FTIR), revealing that the structural bands of the biopolymer of GS-14 were nearly identical to those of the standard PHA, including the characteristic C = O and C-O stretching bands essential for identifying polyesters. This similarity was further validated via liquid chromatography-mass spectrometry, confirming the presence of regularly spaced peaks approximately 60 Da apart, indicating the presence of a repeating monomeric unit, a hallmark feature of polyhydroxyalkanoates (PHAs). Nuclear magnetic resonance (NMR) studies offered additional structural details, pinpointing specific resonances for methyl, methylene, and hydroxyl groups. Differential scanning calorimetry (DSC) analysis illustrated the thermal stability and crystallinity of the biopolymer, providing key insights into its mechanical strength and application potential.

Phylogenetic analysis based on sequencing identified GS-14 as *Bacillus australimaris*, and the sequence was deposited in the NCBI repository, contributing to the broader scientific understanding of PHA-producing bacteria.

To screen media components for maximizing PHA production, a Plackett–Burman experimental design was implemented to determine the influence of various factors on biomass and PHA yield. The analysis revealed that (NH₄)₂SO₄ was critical in promoting higher yields, with statistical validation confirming their significant impact. Although (NH₄)₂SO₄ was initially considered less crucial for PHA accumulation, this study clarified its role in enhancing biomass, confirming the role of a limited nitrogen source in driving PHA production. MgSO₄ was also found to have a notable influence on biomass, underscoring the importance of balanced nutrient provision.

Using Minitab’s statistical insights, a clearer understanding of the factors governing PHA production was achieved, with 2D contour plots illustrating the relationships between variables and their effects on biomass and PHA yield. This study presents promising evidence for the industrial application of *Bacillus australimaris* GS-14 in sustainable bioplastic production, paving the way for further optimization and scale-up strategies in microbial PHA biosynthesis.

To understand the detailed mechanism of PHA induction and bacteria’s absorption of carbon, nitrogen, and phosphorus, it is essential to examine the biochemical pathways and environmental factors that drive these processes. The biosynthesis of PHA involves three key enzymes: acetyl-CoA acetyltransferase (phaA), acetoacetyl-CoA reductase (phaB), and PHA synthase (phaC)^41^.

These enzymes collectively convert acetyl-CoA into PHA, which is stored as intracellular granules. The genes encode these enzymes in the PHA operon (phaCAB), and specific promoters control their expression.

Bacteria typically accumulate PHA under conditions where carbon is in excess, but other essential nutrients, such as nitrogen or phosphorus, are limited. In such scenarios, PHA serves as a carbon and energy reserve. PHA synthase primarily mediates the process, which polymerizes acetoacetyl-CoA into PHA. Nitrogen limitation is one of the critical triggers for PHA accumulation, as the scarcity of nitrogen forces bacteria to redirect the carbon flux towards PHA synthesis, helping them maintain metabolic balance^42^.

Phosphorus uptake is achieved through various mechanisms, including phosphate transporters and the formation of polyphosphate granules. The induction of PHA in bacteria is a complex process, governed by specific enzymes and genetic operons, and is significantly influenced by environmental factors such as nutrient availability and carbon excess. The absorption of carbon, nitrogen, and phosphorus is closely interconnected with these mechanisms, with each nutrient playing a vital role in bacterial metabolism and the production of PHA^43^.

## Future perspectives

Moving forward, future research will focus on several key areas. This includes media optimization via response surface methodology (RSM) to maximize PHA production by *B. australimaris*, scaling up for large-scale PHA production using agricultural waste as the primary substrate, and conducting a technoeconomic analysis to assess the economic feasibility of large-scale PHA production. Furthermore, efforts will be directed toward characterizing the mechanical and thermal properties of the extracted PHA to determine its suitability for various bioplastic applications and investigating the in vitro biocompatibility of the produced PHA for potential biomedical applications. By achieving these objectives, this research has the potential to offer a sustainable alternative to petroleum-based plastics, reduce dependence on fossil fuels, minimize plastic waste generation, provide economic benefits through the utilization of agricultural waste streams, and broaden the applications of bioplastics in various sectors. This study thus paves the way for developing a novel and sustainable approach to PHA production, promoting environmental responsibility and fostering a biobased economy.

## Conclusion

This study successfully isolated and identified a potent PHA-producing bacterial strain, *Bacillus australimaris*, from soil samples utilizing agricultural waste products. The isolate demonstrated promising PHA yield via a cost-effective and eco-friendly approach. Key achievements include the development of a reliable method for isolating PHA-producing bacteria from diverse soil sources, the identification of *Bacillus australimaris* as a high-yield PHA producer using agricultural waste as a carbon source, and the characterization of the extracted PHA polymer via various analytical techniques to confirm its structural identity. Additionally, this study employed a statistical design (Plackett-Burman) to identify crucial media components influencing PHA production.

## Electronic supplementary material

Below is the link to the electronic supplementary material.

Supplementary Material 1

## Data Availability

The datasets generated and/or analysed during the current study are available in the GEN BANK repository, GenBank accession number is SUB14851199 23C110_339_GS PQ569061. A copy of the revised files can be viewed at https://www.ncbi.nlm.nih.gov/nuccore/2846111647.
