# Author Correction: Production, isolation, optimization, and characterization of microbial PHA from *Bacillus australimaris*

**DOI:** 10.1038/s41598-025-02041-8

**Published:** 2025-05-15

**Authors:** Rafwana Ibrahim, Jesil Mathew Aranjani, Navya Prasanna, Avirup Biswas, Prasanna Kumar Reddy Gayam

**Affiliations:** Department of Pharmaceutical Biotechnology, Manipal College of Pharmaceutical Sciences, Manipal Academy of Higher Education, Manipal, 576140 India

Author Correction: *Scientific Reports* 10.1038/s41598-025-92146-x, published online 11 March 2025

In the original version of this Article, the Author contributions section was incomplete.

“Conceptualization and Supervision: Dr. Jesil Mathew A guided the research, conceptualizing the study’s framework and providing critical oversight throughout the project. He played a pivotal role in the study design, ensuring scientific rigor and validating the experimental methodology and outcomes. Experimental Work and Data Curation: Navya Prasanna was responsible for conducting the experimental procedures under the supervision of Avirup. Navya managed the isolation, optimization, and characterization of microbial PHA production from Bacillus australimaris, including laboratory work, data collection, and analysis. Manuscript Writing, Methodology Refinement, and Editing: Rafwana Ibrahim contributed to the manuscript’s development by overseeing the writing and editing process. Additionally, she reworked some of the methodologies to align them with the study’s objectives, ensuring the clarity, coherence, and scientific accuracy of the research presentation.”

now reads:

“Conceptualization and Supervision: Dr. Jesil Mathew A. guided the research, conceptualizing the study’s framework and providing critical oversight throughout the project. He played a pivotal role in the study design, ensuring scientific rigor and validating the experimental methodology and outcomes. Experimental Work and Data Curation: Navya Prasanna was responsible for conducting the experimental procedures under the supervision of Avirup. Navya managed the isolation, optimization, and characterization of microbial PHA production from Bacillus australimaris, including laboratory work, data collection, and analysis. Manuscript Writing, Methodology Refinement, and Editing: Rafwana Ibrahim contributed to the manuscript’s development by overseeing the writing and editing process. Additionally, she reworked some of the methodologies to align them with the study’s objectives, ensuring the clarity, coherence, and scientific accuracy of the research presentation. Prasanna Kumar Reddy Gayam made a significant contribution by critically interpreting the data and modifying the manuscript to align with the required format and standards set by the reviewers. His involvement in refining the analysis and restructuring the manuscript as per the journal’s guidelines constitutes a substantial intellectual contribution.”

In addition, the Acknowledgements section was incomplete.

“The authors extend their heartfelt gratitude to the Manipal Academy of Higher Education for providing the laboratory space and funding necessary for conducting the experiments detailed in this research. Additionally, we would like to acknowledge the invaluable support from late Dr. Alex Joseph during this study, including his assistance with spectral data interpretation.”

now reads,

“The authors extend their heartfelt gratitude to the Manipal Academy of Higher Education for providing the laboratory space and funding necessary for conducting the experiments detailed in this research. The fellowship provided to Prasanna Kumar Reddy Gayam by the Council of Scientific & Industrial Research (CSIR), through the NET fellowship (08/602(0008)/2020-EMR-I) is also acknowledged. Additionally, we would like to acknowledge the invaluable support from the late Dr. Alex Joseph during this study, including his assistance with spectral data interpretation.”

The original Article has been corrected.

