# Supplementary material for: Production, isolation, optimization, and characterization of microbial PHA from Bacillus australimaris
Source: Sci Rep. 2025 Mar 11;15:8395. doi: 10.1038/s41598-025-92146-x (PMC11897369; doi:10.1038/s41598-025-92146-x)
Supplement: Supplementary file 1 — Supplementary Material 1 [file 41598_2025_92146_MOESM1_ESM.docx]

**Production, Isolation, Optimization, and Characterization of Microbial PHA from *Bacillus australimaris***

Rafwana Ibrahim, Dr Jesil Mathew, Navya Prasanna, Avirup Biswas

1. Department of Pharmaceutical Biotechnology, Manipal College of Pharmaceutical Sciences, Manipal Academy of Higher Education, 576140

**SUPPLEMENTARY DATA**

**Description of Column Headers**

1.     **qseqid:** query or source sequence id

2.     **sseqid:** subject or target (e.g., reference genome) sequence id

3.     **pident:** percentage of identical matches

4.     **length:** alignment length

5.     **mismatch:** number of mismatches

6.     **gapopen:** number of gap openings

7.     **qstart:** start of alignment in query

8.     **qend:** end of alignment in query

9.     **sstart:** start of alignment in subject

10. **send:** end of alignment in subject

11. **evalue:** the number of expected hits of similar quality (score) that could be found just by chance.

12. **bitscore:** bit score indication about the statistical significance of an alignment.

**Characterization of PHA**

*Supplementary Figure 1: FTIR of PHA from CS-3 strain*

*Supplementary Figure 2: FTIR of PHA from CS-4 strain*

## *Supplementary Figure 3: FTIR of PHA from CS-5 strain*

## *Supplementary Figure 4: FTIR of PHA from CS-6 strain*

## *Supplementary Figure 5: FTIR of PHA from CS-8 strain*

## *Supplementary Figure 6: FTIR of PHA from CS-9 strain*

*Supplementary Figure 7: FTIR OF PHA from CS-10 strain*

## *Supplementary Figure 8: FTIR of PHA from GS-11 strain*

## *Supplementary Figure 9: FTIR of PHA from GS-12 strain*

## *Supplementary Figure 10: FTIR of PHA from GS-15 strain*

*Supplementary Figure 11: FTIR of PHA from GS-16 strain*

*Supplementary Figure 12: FTIR of PHA from GS-17 strain*

**Sequence Electropherogram(s)**

The sequence data as an Electropherogram for each sequencing reaction can be visualized.


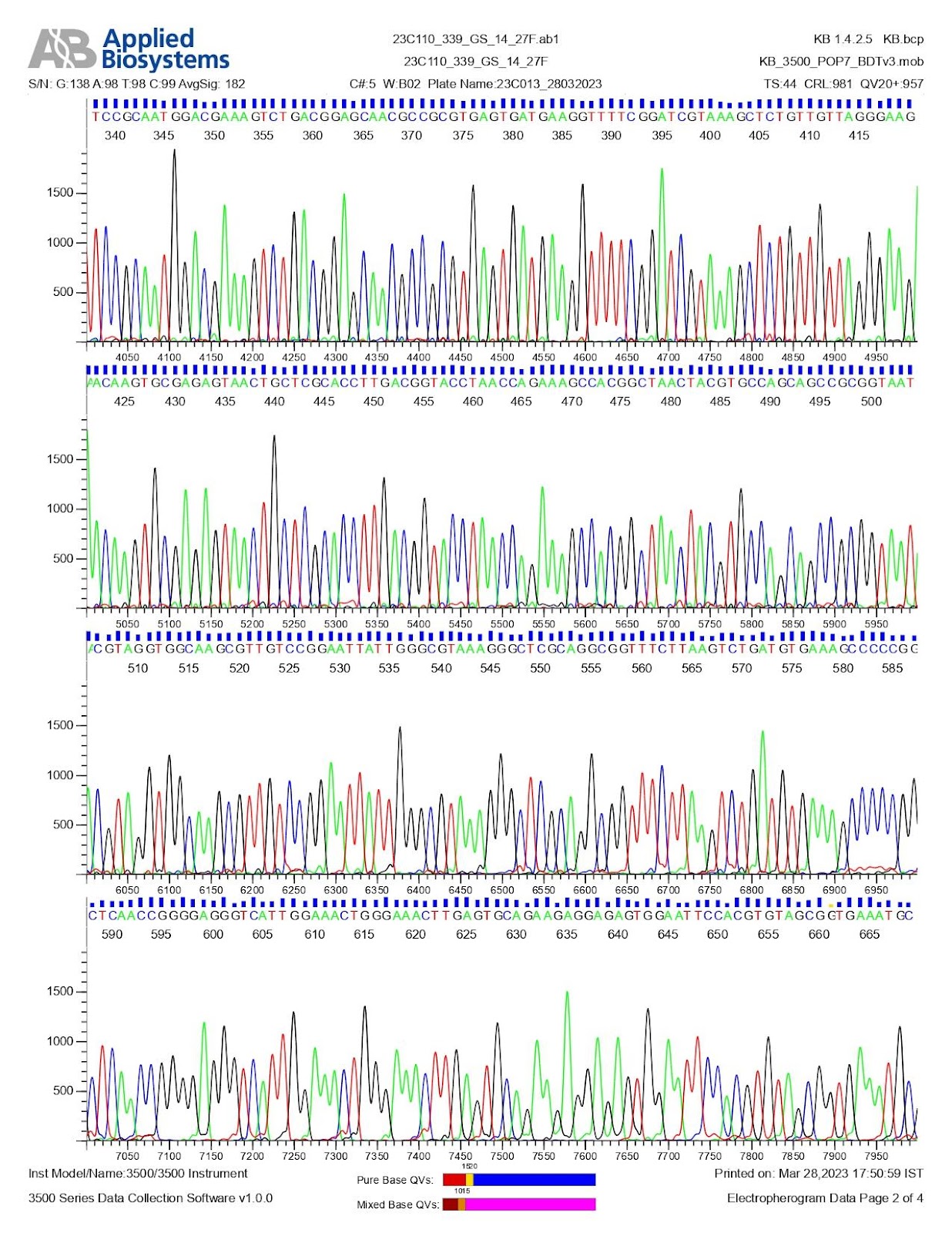


**a)**


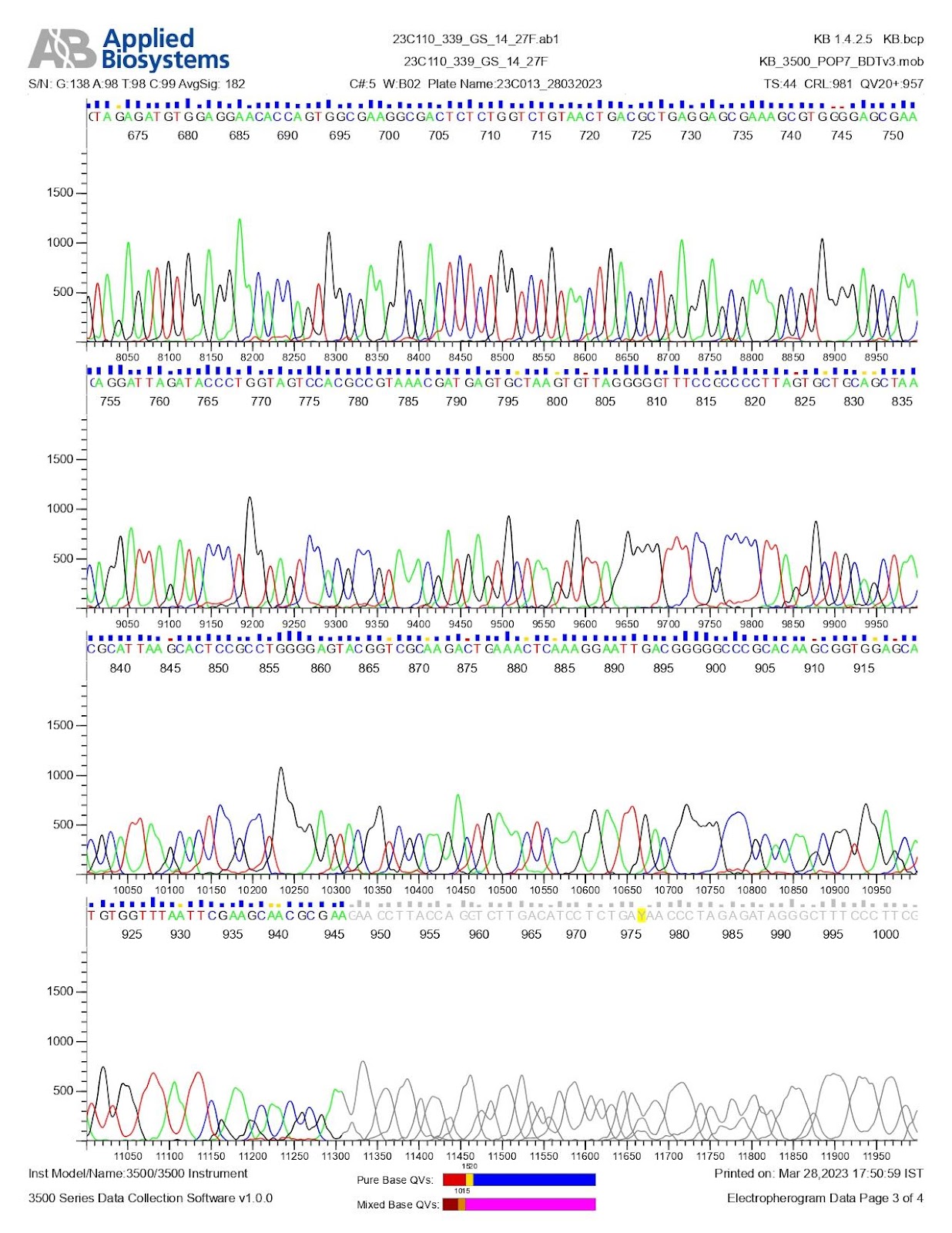
 **b)**


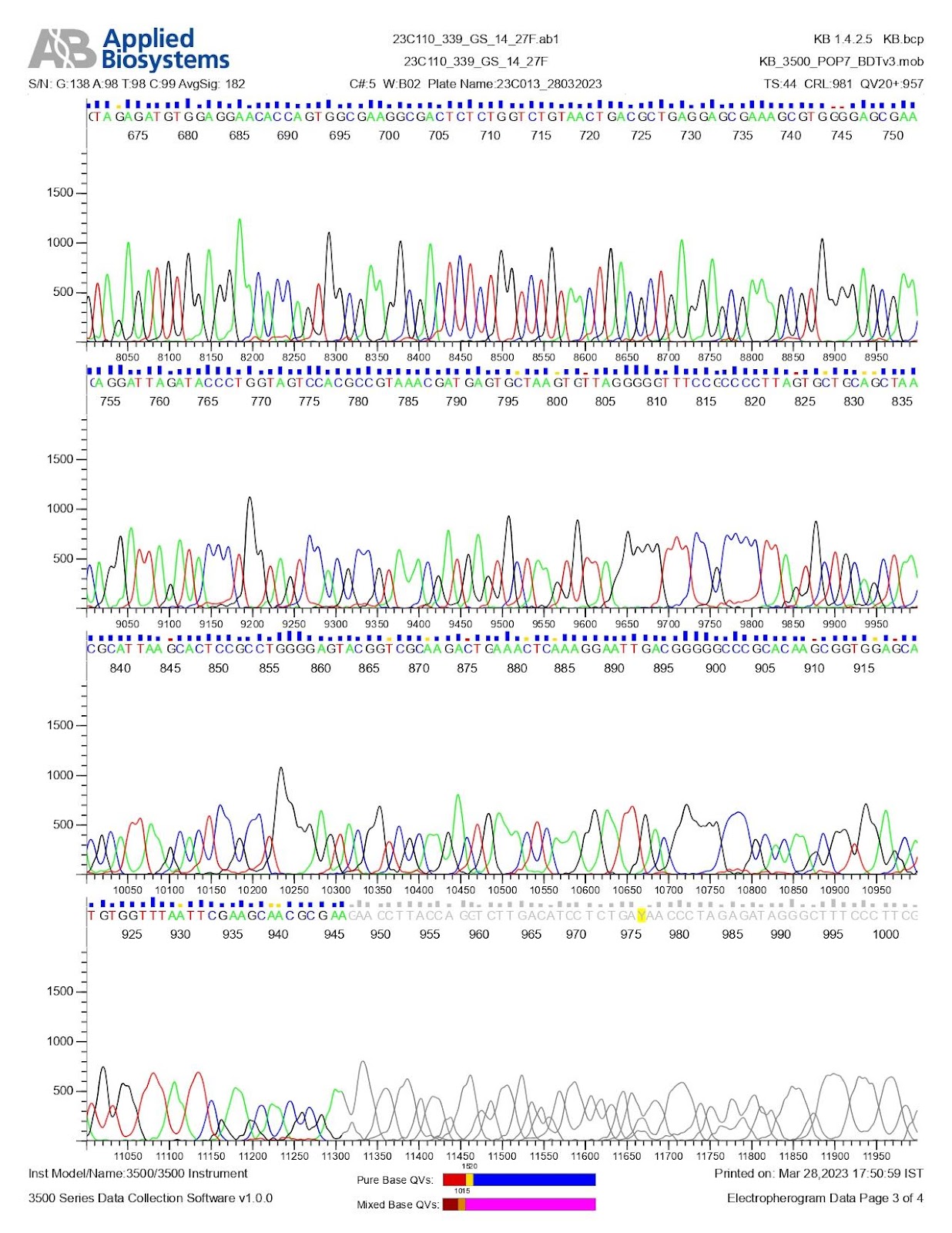


**c)**


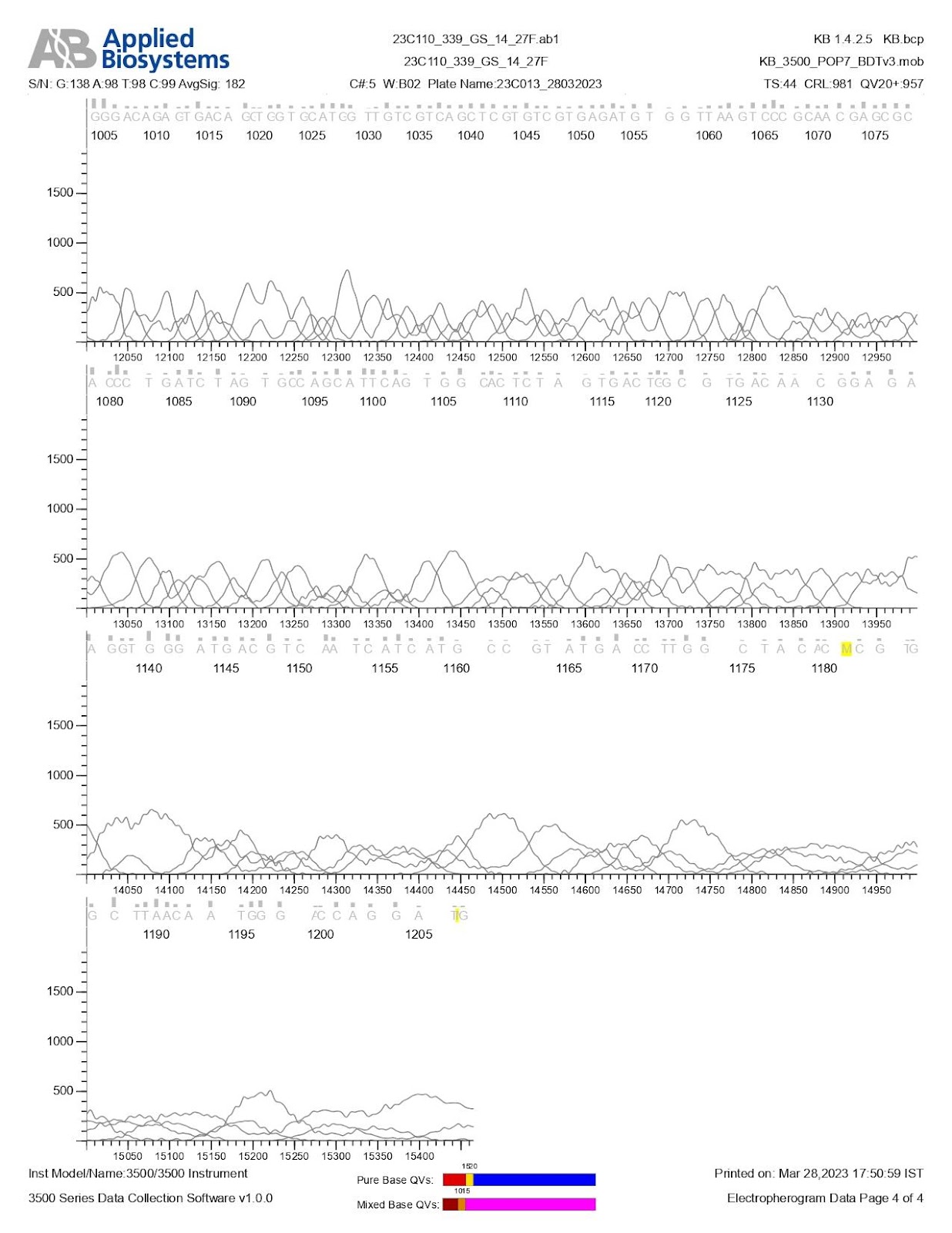


**d)**

**
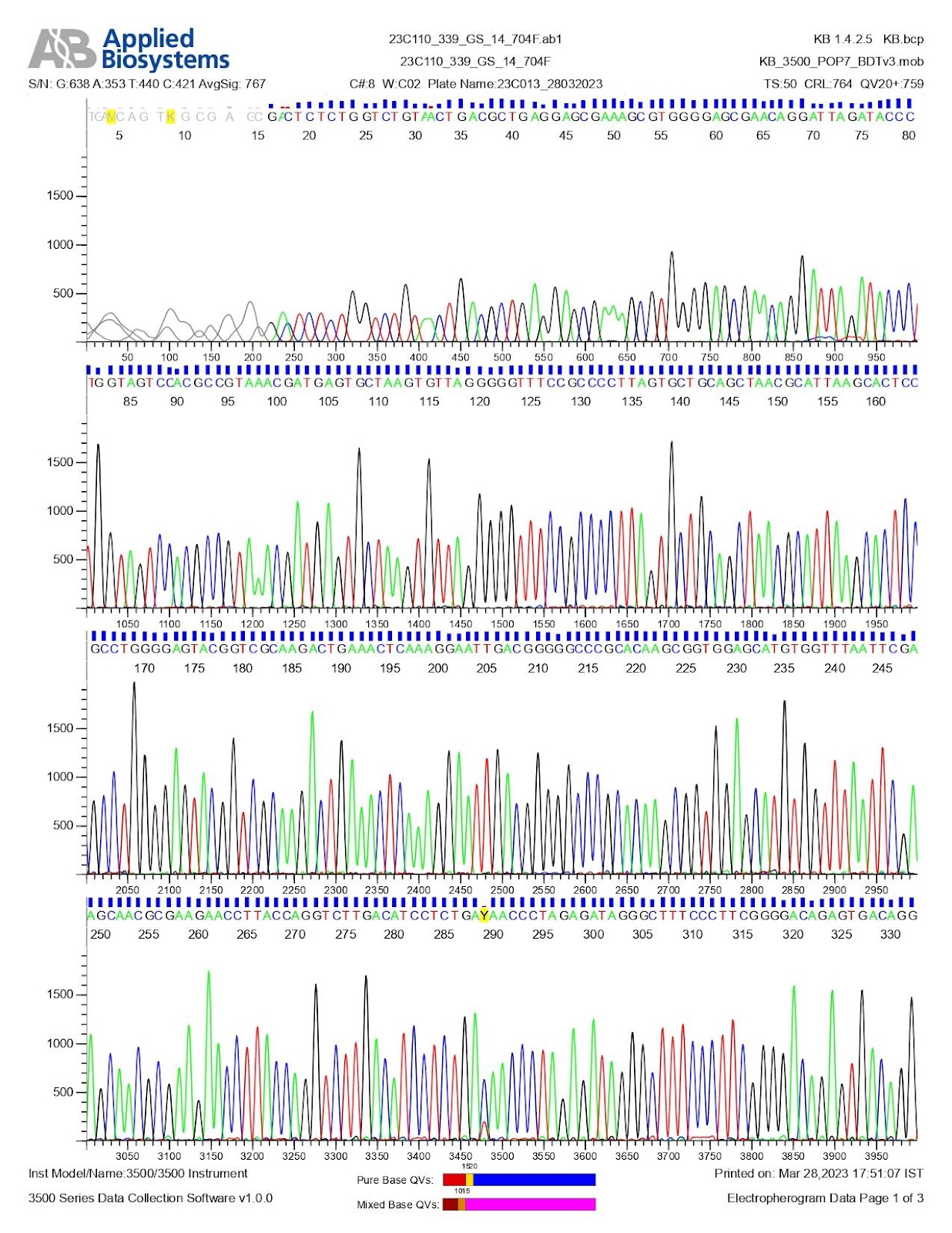
**

**e)**

**
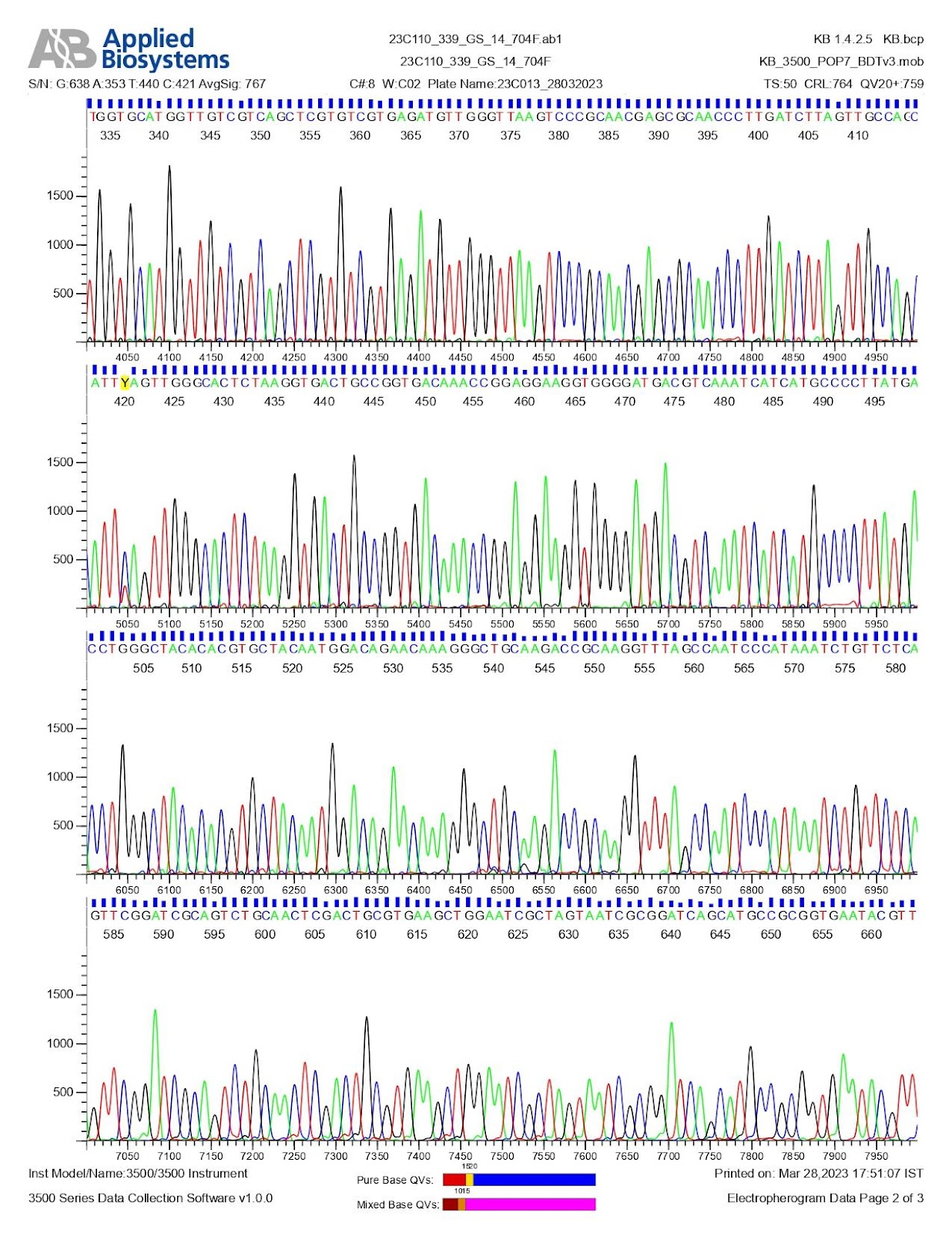
**

**f)**


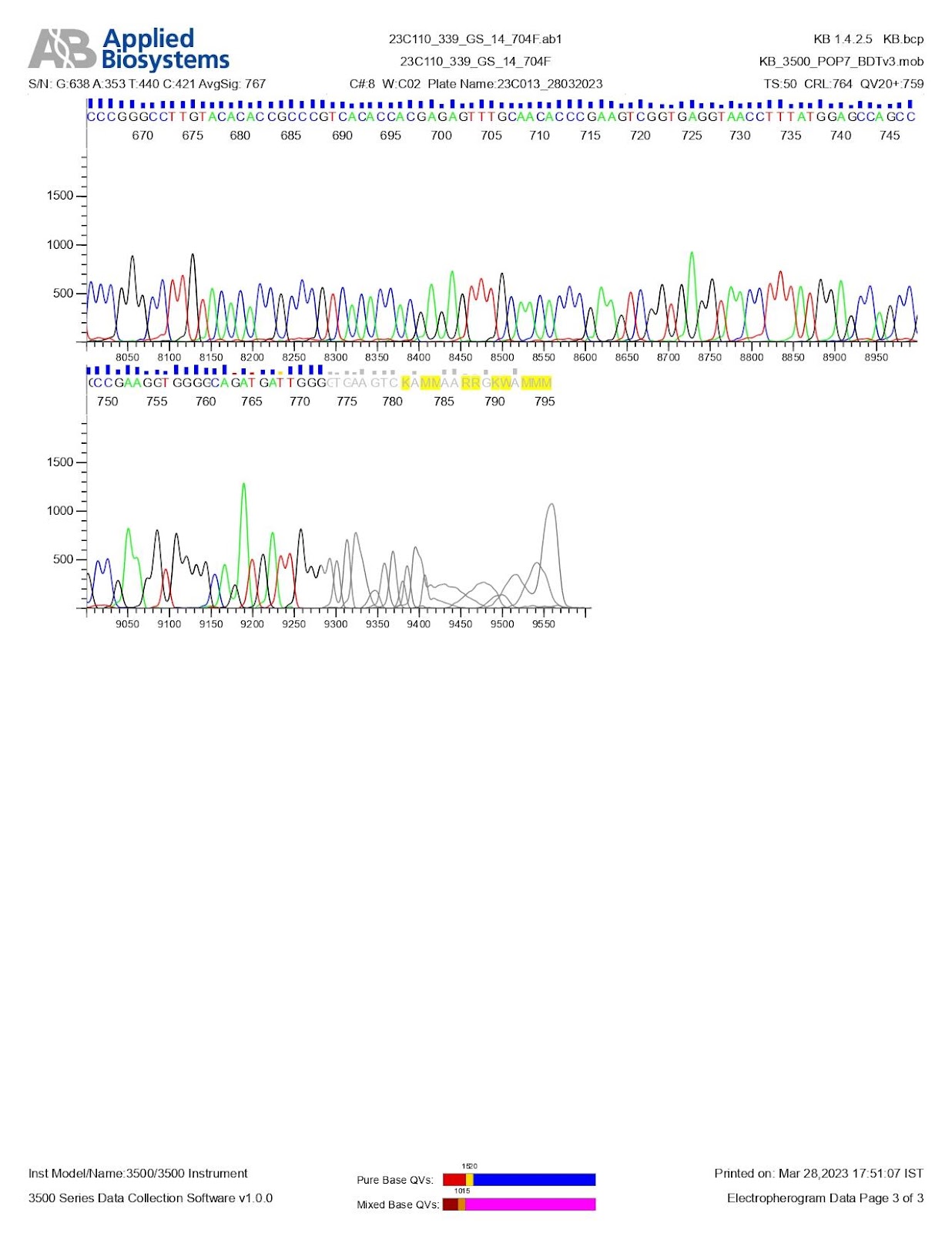


**g)**

Supplementary Figure 13: a, b, c, d, e, f, g: Shows Electropherogram of the isolate.
